# Histone H3K18 Lactylation Promotes the Malignant Progression of Wilms Tumor via a PSRC1/AKT/HIF‐1α Positive Feedback Loop

**DOI:** 10.1002/advs.76579

**Published:** 2026-07-13

**Authors:** Yanping Wang, Hongjie Gao, Bifei Zhang, Xuetian Li, Ding Li, Aihua Cao, Fengyin Sun

**Affiliations:** ^1^ Department of Pediatric Surgery Qilu Hospital of Shandong University Jinan China; ^2^ Department of Pediatrics Qilu Hospital of Shandong University Jinan China

**Keywords:** AKT, H3K18 lactylation, HIF‐1α, positive feedback loop, PSRC1, Wilms tumor

## Abstract

Nephroblastoma, also known as Wilms tumor (WT), is the most common pediatric renal malignancy. Current treatment regimens exhibit limited efficacy in high‐risk patients and are associated with long‐term adverse effects. Through gene set enrichment analysis (GSEA) and validation using clinical specimens, we identified aberrant glycolytic activity in WT, which drives lactate accumulation. Lactate is not merely a metabolic byproduct but also a critical epigenetic regulator that mediates histone lactylation; however, its functional role in WT remains uncharacterized. In this study, we found that the hyperlactate microenvironment induced by abnormal glycolysis in WT significantly upregulates the level of histone H3K18 lactylation (H3K18la). Functional experiments confirmed that histone lactylation promotes WT cell proliferation and migration. Mechanistically, H3K18la, catalyzed by its “writer” p300, directly marks the promoter region of the downstream target gene PSRC1 and transcriptionally activates its expression. PSRC1 then acts as a key “molecular competitor,” binding to AKT in a competitive manner with the phosphatase PTEN. This interaction relieves PTEN‐mediated dephosphorylation inhibition of AKT, thereby activating the AKT/mTOR signaling pathway and enhancing the protein stability of its downstream effector HIF‐1α. Concurrently, HIF‐1α functions as a transcription factor to directly bind to the PSRC1 promoter, synergizing with H3K18la to further amplify PSRC1 transcription. In summary, this study identifies a positive feedback loop: “H3K18la→PSRC1→AKT/mTOR→HIF‐1α→PSRC1.” This mechanism dynamically links tumor metabolic abnormalities, histone modifications, and key oncogenic signaling pathways, which collectively drive the malignant progression of WT. These findings provide a novel theoretical basis for the stratified diagnosis and targeted therapy of WT.

## Graphical abstract

In nephroblastoma, aberrant glycolysis drives lactate accumulation, which elevates histone H3K18 lactylation via p300. Lactylation of the PSRC1 promoter activates its transcription. PSRC1 competitively binds AKT, relieving PTEN‐mediated inhibition and triggering AKT/mTOR/HIF‐1α signaling. HIF‐1α further upregulates PSRC1, forming a positive feedback loop that links metabolic reprogramming, epigenetic regulation, and oncogenic signaling to promote tumor progression.

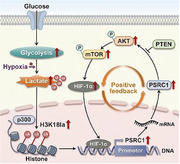

## 1 Introduction

Nephroblastoma, also known as Wilms tumor (WT), is the most prevalent pediatric renal malignancy [1]. While over 90% of pediatric patients are diagnosed with favorable‐histology Wilms tumor (FHWT), the conventional treatment regimens yield variable therapeutic outcomes across different FHWT patient cohorts. Furthermore, 5%–10% of high‐risk WT cases—encompassing newly diagnosed metastatic blastemal‐type, diffuse anaplastic, chemotherapy‐refractory, or multiply relapsed subtypes—remain associated with a survival rate of less than 50%, even following multimodal comprehensive therapy [2]. Even among WT survivors, intensive radiochemotherapy often results in severe long‐term adverse effects, which substantially undermine their quality of life [3]. Thus, identifying novel and promising anticancer targets to facilitate individualized treatment strategies is pivotal for advancing the current therapeutic framework for WT.

“Metabolic reprogramming” is a well‐recognized hallmark of cancer. In numerous tumor types, cancer cells display a distinct metabolic phenotype—they preferentially catabolize glucose to lactate even under oxygen‐replete conditions, a phenomenon termed the Warburg effect [4]. This metabolic shift culminates in substantial lactate accumulation within the tumor microenvironment. A metabolomics‐based investigation has identified prominent metabolic discrepancies between WT tissues and normal renal tissues, with elevated lactate levels being a notable signature [5]. A retrospective cohort study further demonstrated that higher lactate dehydrogenase (LDH) activity serves as an independent risk factor for predicting increased recurrence risk in pediatric WT patients [6]. Our team's previously published findings have also characterized lactate metabolism‐associated long non‐coding RNAs (lncRNAs) that hold prognostic value for survival outcomes in WT [7]. Collectively, these discoveries preliminarily underscore the potential driving role of aberrant lactate metabolism in the initiation and progression of WT.

In WT, dysregulated histone modifications are increasingly acknowledged as pivotal contributors to tumorigenesis and progression [8]. Among these epigenetic alterations, histone acetylation has been the most extensively investigated. The acetyltransferase p300/CBP, acting as a canonical epigenetic “writer”, can enhance the endogenous expression of WT1—a classical oncogene in WT—thereby fueling tumorigenic processes [9, 10]. Preclinical and clinical studies have confirmed that the overexpression of multiple histone deacetylase (HDAC) isoforms promotes WT cell proliferation via transcriptional silencing of tumor suppressor genes [11, 12]. Moreover, HDAC inhibitors have shown therapeutic potential in WT by downregulating WT1 expression while simultaneously targeting key oncogenic signaling pathways [13, 14].

Lactate, once regarded merely as a metabolic byproduct or an agent inducing cellular fatigue, is now recognized as a versatile small‐molecule metabolite that functions as an energy substrate, signaling molecule, and epigenetic regulator [15, 16]. In 2019, Zhao et al. first identified histone lactylation as a novel epigenetic modification, which acts as a molecular switch to directly modulate gene transcription on chromatin [16]. Since this landmark discovery, a growing body of evidence has revealed that histone lactylation is involved in multiple cancer hallmarks, including immune evasion, metabolic reprogramming, and therapeutic resistance, thus facilitating the malignant progression of various tumors [17]. Similar to histone acetylation, histone lactylation is also an enzyme‐dependent epigenetic process. Intriguingly, histone lactylation and acetylation share a partially overlapping regulatory enzyme repertoire—p300/CBP and HDAC1‐3/SIRT1‐3 also serve as the “writers” and “erasers” for histone lactylation, respectively [18, 19, 20]. This striking mechanistic overlap strongly implies that histone lactylation may play a vital role in WT pathogenesis. However, its molecular mechanisms and therapeutic potential remain unclear, requiring further investigation.

In this study, we report that histone lactylation modification—specifically the level of histone H3 lysine 18 lactylation (H3K18la)—is selectively elevated in WT tissues and functionally drives tumor progression. Mechanistically, p300‐mediated H3K18la directly targets the PSRC1 promoter to activate its transcription. PSRC1 then acts as a critical “molecular competitor” by competitively binding to AKT, thereby abrogating the inhibitory effect of the phosphatase PTEN on AKT dephosphorylation. This event triggers sustained activation of the core oncogenic AKT/mTOR/HIF‐1α signaling pathway. In turn, cascade‐induced accumulated HIF‐1α functions as a transcription factor to directly bind to the PSRC1 promoter region, synergizing with H3K18la to further amplify PSRC1 transcription. Collectively, these events constitute a self‐reinforcing feedback loop that amplifies oncogenic signaling. This research pioneers the elucidation of a novel regulatory mechanism in WT. These findings may facilitate molecular stratification and precision therapy for WT.

## 2 Materials and Methods

### 2.1 Clinical Human Specimens

This study utilized 52 pairs of Wilms tumor (WT) tissues and matched adjacent normal tissues collected from Qilu Hospital of Shandong University. All clinical samples were obtained from surgical resection specimens, and none of the pediatric patients had undergone radiotherapy, chemotherapy, or other anti‐tumor treatments prior to surgery. The use of all specimens was approved by the Institutional Research Ethics Committee of Qilu Hospital (Approval No.: KYLL‐2026(ZM)‐1131). All specimens and data were de‐identified prior to analysis to protect patient privacy.

### 2.2 RNA‐seq and ChIP‐seq Data Sources

RNA‐seq datasets and corresponding clinical information from 126 WT patients and 6 normal children were retrieved from the Therapeutically Applicable Research to Generate Effective Treatments (TARGET) database. Additional RNA‐seq datasets of WT and normal tissues (GSE197047; GSE66405), H3K18la ChIP‐seq datasets (GSE156675; GSE295142; GSE245990), and p300 ChIP‐seq datasets (GSE304259) were downloaded from the Gene Expression Omnibus (GEO) database.

### 2.3 Bioinformatics Analysis

Based on the TARGET‐WT dataset and GEO‐derived WT datasets, we conducted the following bioinformatic analyses: Gene Set Enrichment Analysis (GSEA) was performed using relevant gene sets from the Molecular Signatures Database (MSigDB) [21]. Differential gene expression analysis, Kaplan‐Meier survival analysis, Receiver Operating Characteristic (ROC) curve plotting, univariate/multivariate Cox proportional hazards regression analysis, and nomogram construction were implemented using the SangerBox 3.0 online platform [22].

ChIP‐seq data were analyzed using the GEO database and Integrative Genomics Viewer (IGV) software (v 2.19.6). PSRC1‐interacting proteins were predicted via the BioGRID database (v 5.0) [23]. Binding sequences of HIF‐1α in the PSRC1 promoter region were predicted using the JASPAR database [24]. Functional domains of PSRC1 were delineated via the UniProt database to guide truncation plasmid construction [25].

Molecular Docking: All protein structures were retrieved from the UniProt database. Proteins were processed using the Molecular Operating Environment (MOE 2019.1) software platform, including water and ion removal, protonation, supplementation of missing atoms and groups, and energy minimization. HADDOCK software was used for molecular docking, with proteins set as rigid bodies and docking contact sites covering the entire protein surface. After docking, the conformation with the lowest binding energy was selected and visualized using PyMOL software.

### 2.4 Glycolytic Metabolite Detection

Glucose assay kits (glucose oxidase method) and lactate assay kits were purchased from Nanjing Jiancheng Bioengineering Institute. Assays were performed, and results were analyzed strictly following the manufacturer's protocols, with three biological replicates per group.

### 2.5 Cell Culture and Drug Treatment

HEK‐293T cells (Cell Bank, Chinese Academy of Sciences), WiT49 cells (The Affiliated Hospital of Qingdao University), and WT‐CLS1 cells (Chongqing Medical University) were cultured in Dulbecco's Modified Eagle Medium (DMEM) supplemented with 10% fetal bovine serum (FBS; Gibco) and 100 U/mL penicillin–streptomycin, under humidified conditions of 37°C and 5% CO_2_. All cell lines were authenticated and confirmed to be free of mycoplasma contamination. For drug treatment experiments, the concentration and treatment duration of all drugs were determined via preliminary experiments. Detailed information on drug sources is provided in Table S5.

#### 2.5.1 Metabolic Modulator Treatment

Sodium lactate (NaLa) was used to increase intracellular lactate levels at concentrations of 0, 2.5, 5, 10, and 20 mM. Glycolysis inhibitors 2‐deoxy‐D‐glucose (2‐DG) and oxamate (OXA) were used to reduce lactate production at concentration gradients of 0, 2.5, 5, 10, and 20 mM. All drugs were pre‐dissolved in phosphate‐buffered saline (PBS) or sterile water to prepare stock solutions, and the treatment duration was 24–48 h.

#### 2.5.2 Hypoxia Mimetic Treatment

Cobalt chloride (CoCl_2_) powder was dissolved in sterile water to prepare a stock solution. To simulate a hypoxic environment and induce HIF‐1α expression, cells were cultured in medium containing 100 µM CoCl_2_ for 12–24 h.

#### 2.5.3 Signaling Pathway Modulator Treatment

The AKT agonist SC79 and AKT inhibitor MK2206 were dissolved in dimethyl sulfoxide (DMSO) to prepare stock solutions. Cells were treated with 20 µM SC79 or 1 µM MK2206 for 24–48 h, respectively. The control group received an equal volume of DMSO (< 0.1%).

#### 2.5.4 Protein Half‐Life Detection

The protein synthesis inhibitor cycloheximide (CHX) was dissolved in DMSO. Cells were treated with 100 µg/mL CHX, and total protein samples were collected at 0, 30, 60, and 90 min post‐treatment for subsequent experiments.

### 2.6 Plasmid Construction and Cell Transfection

Short hairpin RNA (shRNA) sequences targeting PSRC1, p300, and HIF1A were cloned into the pLKO.1 vector; specific sequences are listed in Table S1. The pLKO.1 vector encoding a scrambled shRNA sequence was used as a negative control. PSRC1 overexpression plasmids, truncation plasmids, and epitope‐tagged plasmids (Flag‐, MYC‐, or HA‐tagged) were purchased from Miaoling Biotechnology Co., Ltd., with pCDNA3.1 as the backbone vector.

Transient transfection for short‐term experiments was performed using Lipo8000 transfection reagent (Beyotime, China). To establish stable cell lines with PSRC1 knockdown or overexpression for in vivo experiments, lentiviral packaging was conducted by co‐transfecting HEK‐293T cells with a three‐plasmid system (target vector, psPAX2, and pMD2.G). WT cells were infected with the collected viral supernatant and selected continuously with puromycin to obtain stable cell lines.

### 2.7 Immunohistochemistry (IHC)

Clinical tissues were paraffin‐embedded and sectioned by Servicebio Technology Co., Ltd. After deparaffinization and rehydration, antigen retrieval was conducted in sodium citrate or Tris‐EDTA buffer (Solarbio, China) via high‐temperature heating. Sections were sequentially blocked with peroxidase blocker (ZSGB‐BIO, China) and 5% BSA, then incubated with primary antibodies overnight at 4°C, followed by HRP‐conjugated secondary antibody incubation. DAB staining and hematoxylin counterstaining were performed prior to dehydration, clearing, and mounting. Slides were imaged with an Olympus VS200 digital slide scanner. Detailed antibody information is listed in Table S4.

The staining intensity was scored on a 0–3 scale: 0 (achromatic), 1 (light yellow), 2 (pale brown), and 3 (sepia). The percentage of positive‐stained cells was scored separately: 0%–25% (1 point), 26%–50% (2 points), 51%–75% (3 points), and 76%–100% (4 points). The final IHC score was calculated by multiplying the intensity score by the positive percentage score.

### 2.8 Western Blot (WB)

Cell and tissue samples were lysed with RIPA buffer containing 1 mM PMSF (Beyotime, China); phosphatase inhibitors were supplemented for phosphorylated protein extraction. Protein samples were mixed with SDS‐PAGE loading buffer and boiled for denaturation. Subsequently, proteins were separated by 4–12%/10% SDS‐PAGE and transferred to PVDF membranes (Millipore, USA). Membranes were rinsed with TBST buffer, blocked with 5% skim milk, and then incubated with primary antibodies at 4°C overnight, followed by HRP‐conjugated secondary antibody incubation. Protein bands were visualized using ECL chemiluminescence reagent (China). All antibodies used are listed in Table S4.

### 2.9 Real‐Time Quantitative PCR (RT‐qPCR)

Total RNA was extracted from cell samples using a Total RNA Extraction Kit (Accurate Biotechnology, China) and reverse‐transcribed into complementary DNA (cDNA) using a Reverse Transcription Kit with gDNA Eraser (ABclonal, China). Real‐Time quantitative PCR (RT‐qPCR) was performed using Universal SYBR Green Fast qPCR Mix (ABclonal, China). Relative gene expression levels were normalized to ACTB (β‐actin) and calculated using the 2^−ΔΔCt^ method. Primer sequences were synthesized by Sangon Biotech (Shanghai, China) and are listed in Table S2.

### 2.10 Chromatin Immunoprecipitation Followed by qPCR (ChIP‐qPCR)

Pretreated cells were cultured in 10 cm dishes until 90%–100%, followed by formaldehyde cross‐linking and glycine termination. Chromatin was fragmented via micrococcal nuclease digestion combined with sonication. After low‐temperature centrifugation, the supernatant was harvested, with a portion reserved as the Input sample. The remaining supernatant was pre‐cleared with Protein A/G magnetic beads, then incubated with anti‐H3K18la, anti‐p300, anti‐HIF‐1α, or control IgG antibodies overnight. Magnetic beads were subsequently added to enrich antibody‐protein‐DNA complexes. Samples were subjected to cross‐link reversal and proteinase K digestion, and DNA was purified using a magnetic bead‐based purification kit (Beyotime, China). Purified DNA was analyzed by qPCR. PSRC1 promoter primer sequences and relevant antibodies are listed in Tables S3 and S4, respectively.

### 2.11 CCK‐8 Cell Proliferation Assay

Cells were seeded in 96‐well plates. CCK‐8 reagent (GLPBIO, USA) was added at 0, 24, 48 and 72 h after seeding. Following incubation (10 µL CCK‐8 in 100 µL medium per well), absorbance at 450 nm was detected with a microplate reader to plot cell growth curves. All experiments were performed in triplicate with three technical replicates per group.

### 2.12 Transwell Cell Migration Assay

Transwell chambers with 8 µm pores (Millipore, USA) were used for migration detection. Cells were resuspended in serum‐free medium and seeded into the upper chamber, while medium containing 20% FBS was added to the lower chamber. After incubation, migrated cells were fixed with methanol and stained with crystal violet. Non‐migrated cells on the membrane upper surface were wiped off, and migrated cells were photographed and quantified using ImageJ software.

### 2.13 Wound Healing Assay

Pretreated cells were cultured in 6‐well plates until 90%–100%. A sterile pipette tip was used to create uniform scratches, followed by PBS washing and replacement with 2% FBS low‐serum medium. Cell migration was imaged at 0, 24, and 48 h under an inverted microscope, and the wound closure rate was quantified using ImageJ software.

### 2.14 Co‐Immunoprecipitation (Co‐IP)

Pretreated cells were lysed on ice in IP lysis buffer containing protease inhibitors. After centrifugation at 4°C, the supernatant was harvested and divided into Input, IgG control, and IP groups. For endogenous Co‐IP, samples were incubated with target antibodies or IgG control together with Protein A/G magnetic beads (MCE, China) at 4°C overnight. For exogenous Co‐IP, cells were first transfected with epitope‐tagged plasmids, followed by incubation with Flag, MYC, or HA magnetic beads (MCE, China) overnight. After incubation, beads were washed three times with IP wash buffer, and co‐precipitated proteins were eluted and analyzed by WB.

### 2.15 Mass Spectrometry (MS) Analysis

To identify proteins that interact with PSRC1, WiT49 cells were transfected with a plasmid encoding FLAG‐tagged PSRC1. After 48 h, proteins were immunoprecipitated using anti‐FLAG magnetic beads. The immunoprecipitated complexes were separated by SDS‐PAGE and stained with Coomassie Brilliant Blue. Target gel bands were excised and sent to LC‐Bio Technology Co., Ltd. for liquid chromatography‐tandem mass spectrometry (LC‐MS/MS) analysis.

### 2.16 Immunofluorescence (IF)

Logarithmic‐phase WT cells were seeded onto glass‐bottom culture dishes at 30%–40% confluence. After attachment, cells were fixed, permeabilized with Triton X‐100, and blocked with 5% BSA. Samples were then incubated with primary antibodies overnight at 4°C. Primary antibodies from different hosts (mouse/rabbit) are detailed in Table S4. After TBST washing, cells were incubated with fluorescent secondary antibodies in the dark. Nuclei were stained with DAPI‐containing anti‐fade mounting medium (Beyotime, China), and images were acquired using a laser scanning confocal microscope.

### 2.17 Animal Models

Prior to the experiment, mice in each group had already been randomized. All animal experiments were approved by the Experimental Animal Ethics Committee of Qilu Hospital, Shandong University (Approval No.: DWLL‐2024‐312).

#### 2.17.1 Xenograft Tumor Model

Male BALB/c nude mice (4–6 weeks old) were purchased from Vital River Laboratory Animal Technology Co., Ltd. (Beijing, China) and housed in the Experimental Animal Center of Qilu Hospital, Shandong University, under specific pathogen‐free (SPF) conditions. WiT49 cells stably transfected with the pCDNA3.1 vector (control) or PSRC1 overexpression plasmid (OE‐PSRC1) were prepared and pretreated with oxamate according to experimental grouping.

Mice were randomly divided into groups (*n* = 5 per group) and subcutaneously injected with 2 × 10^6^ pretreated WiT49 cells per mouse. Cells were resuspended in 100 µL of pre‐cooled PBS mixed with Matrigel (1:1). For the oxamate treatment groups, mice received daily intraperitoneal injections of oxamate (500 mg/kg body weight, dissolved in sterile PBS) starting from the day of inoculation and continuing for the duration of the 25‐day experiment. Control mice received an equal volume of sterile PBS via intraperitoneal injection. Subcutaneous tumor volume and mouse body weight were measured every 5 days. Tumor volume was calculated using the formula: V = W^2^ × L / 2 (W: width, L: length). Mice were euthanized at the endpoint, and tumors were dissected and weighed.

#### 2.17.2 Lung Metastasis Model

For lung metastasis assays, luciferase‐transduced WiT49 cells (1 × 10^6^ cells per mouse) were intravenously administered via the lateral tail vein into male BALB/c nude mice (6–8 weeks old). Body weights were recorded every 2 days, and in vivo bioluminescence imaging was used to quantify pulmonary metastases at the experimental endpoint. Lungs were excised, photographed, and processed for HE staining to confirm and quantify metastatic nodules histologically.

### 2.18 Dual‐Luciferase Reporter Assay

The wild‐type PSRC1 promoter region containing the predicted HIF‐1α binding site was cloned into the pGL3‐Basic vector. An HRE‐mutated construct was generated by site‐directed mutagenesis of the core hypoxia response element (5′‐CGTG‐3′ → 5′‐AAAA‐3′). WiT49 and WT‐CLS1 cells were co‐transfected with the indicated promoter constructs and pRL‐TK (Renilla luciferase internal control) using Lipo8000. Firefly and Renilla luciferase activities were measured using the Dual‐Luciferase Reporter Assay System, and relative luciferase activity was calculated as the Firefly/Renilla ratio.

### 2.19 Kits and Reagents

All experimental kits and reagents used in this study are detailed in Table S5.

### 2.20 Statistical Analysis

Bioinformatic statistical analyses and graphing were performed using R software (version 4.2.1) and the SangerBox 3.0 online platform. Experimental data were analyzed and graphed using ImageJ software and GraphPad Prism software (version 10.0). Blinding was not used in this experiment. The sample sizes were *n* = 5 per group for animal experiments and three biological replicates per group for cell experiments, which meet the generally accepted statistical power standards in this field. Comparisons between two groups were analyzed using Student's t‐test, and comparisons among multiple groups were performed using one‐way analysis of variance (ANOVA) followed by Tukey's post‐hoc test. Most datasets in this study met the normality assumption. For the few datasets that did not meet the normality assumption, we used appropriate non‐parametric tests. A two‐tailed p‐value < 0.05 was considered statistically significant, with significance levels indicated as ^*^ *p* < 0.05, ^**^ *p* < 0.01, ^***^ *p* < 0.001, ^****^ *p* < 0.0001, and ns (not significant) for *p* ≥ 0.05.

## 3 Results

### 3.1 Elevated Lactate Levels Promote H3K18la and Drive Malignant Progression in WT

**FIGURE 1:**
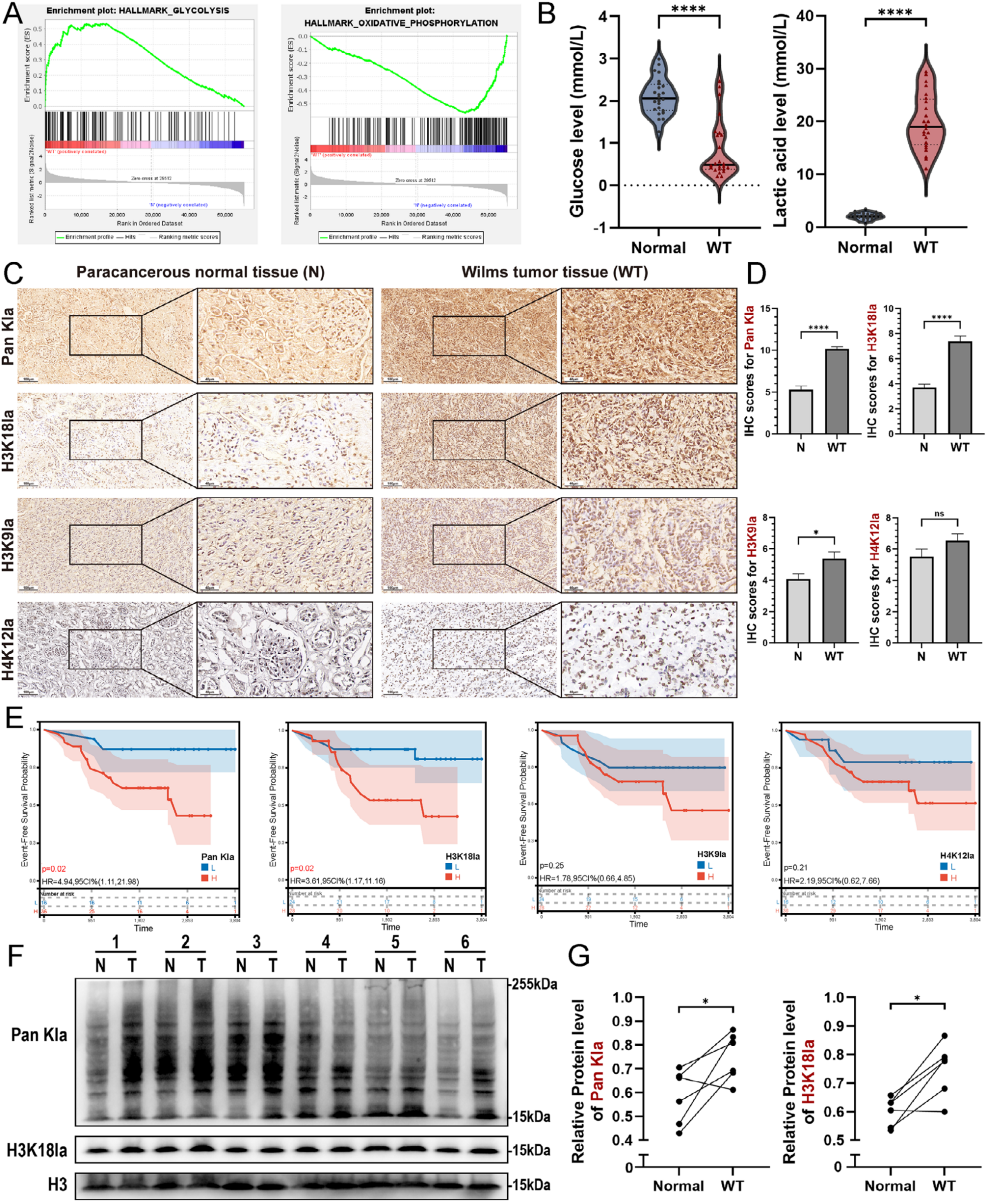
Lactate accumulation induces Pan Kla and H3K18la and correlates with poor prognosis in Wilms tumor tissues. **(A)** GSEA of glycolysis and oxidative phosphorylation pathways in Wilms tumor (WT, *n* = 126) and normal (N; *n* = 6) groups using datasets from the TARGET database. (**B)** Glucose and lactate levels in WT tissues (*n* = 24) and adjacent normal tissues (*n* = 6). (**C, D)** Representative IHC images (C) and IHC scores (D) of Pan Kla, H3K18la, H3K9la, and H4K12la in WT tissues (*n* = 52) and adjacent normal tissues (*n* = 52). (**E)** K‐M event‐free survival (EFS) curves of WT patients stratified by Pan Kla, H3K18la, H3K9la, and H4K12la expression levels (*n* = 52). (**F, G)** WB images (F) and quantitative results (G) of Pan Kla and H3K18la expression in WT tissues (*n* = 6) and adjacent normal tissues (*n* = 6). Data are presented as mean ± SD. ^*^ *p* < 0.05, ^**^ *p* < 0.01, ^***^ *p* < 0.001, ^****^ *p* < 0.0001.

Gene set enrichment analysis (GSEA) was performed on Wilms tumor (WT) datasets retrieved from the TARGET and GEO databases. The results demonstrated that glycolytic metabolic pathways and their associated signaling cascades were markedly more activated in the WT group than in the normal (N) group, whereas the oxidative phosphorylation pathway exhibited higher activity in normal tissues than in tumor tissues (Figure 1A; Figure S1A). This metabolic reprogramming is likely to induce substantial lactate accumulation in tumor tissues, thereby providing a substrate that facilitates histone lactylation modification. To verify this hypothesis, we collected clinical specimens of WT tissues and paired adjacent normal tissues and measured the metabolic levels of glucose and lactate. Consistent with the bioinformatic findings, tumor tissues showed significantly lower glucose content than adjacent normal tissues, whereas lactate levels displayed the opposite trend (Figure 1B).

Previous studies have documented that multiple lysine residues on histones are susceptible to lactylation (Kla) modification [16]. Among the 28 identified histone lactylation sites, H3K18la, H3K9la, and H4K12la have been reported to be most closely correlated with malignant tumor progression [17]. We thus evaluated the expression patterns of these three lactylation sites in clinical samples. Immunohistochemistry (IHC) assays of 52 pairs of WT and adjacent normal tissue specimens revealed that pan‐lysine lactylation (Pan Kla) and histone H3K18 lactylation (H3K18la) exhibited the most prominent upregulation in tumor tissues (Figure 1C,D). Furthermore, survival analyses integrating IHC scores with clinical follow‐up data of patients indicated that only the high‐expression and low‐expression groups of Pan Kla and H3K18la exhibited a statistically significant difference in event‐free survival (EFS) (*n* = 52; *p* < 0.05) (Figure 1E). On the basis of these observations, we focused on H3K18la for subsequent investigations, and further confirmed the elevated expression of Pan Kla and H3K18la in WT tissues via WB analysis (Figure 1F,G).

**FIGURE 2:**
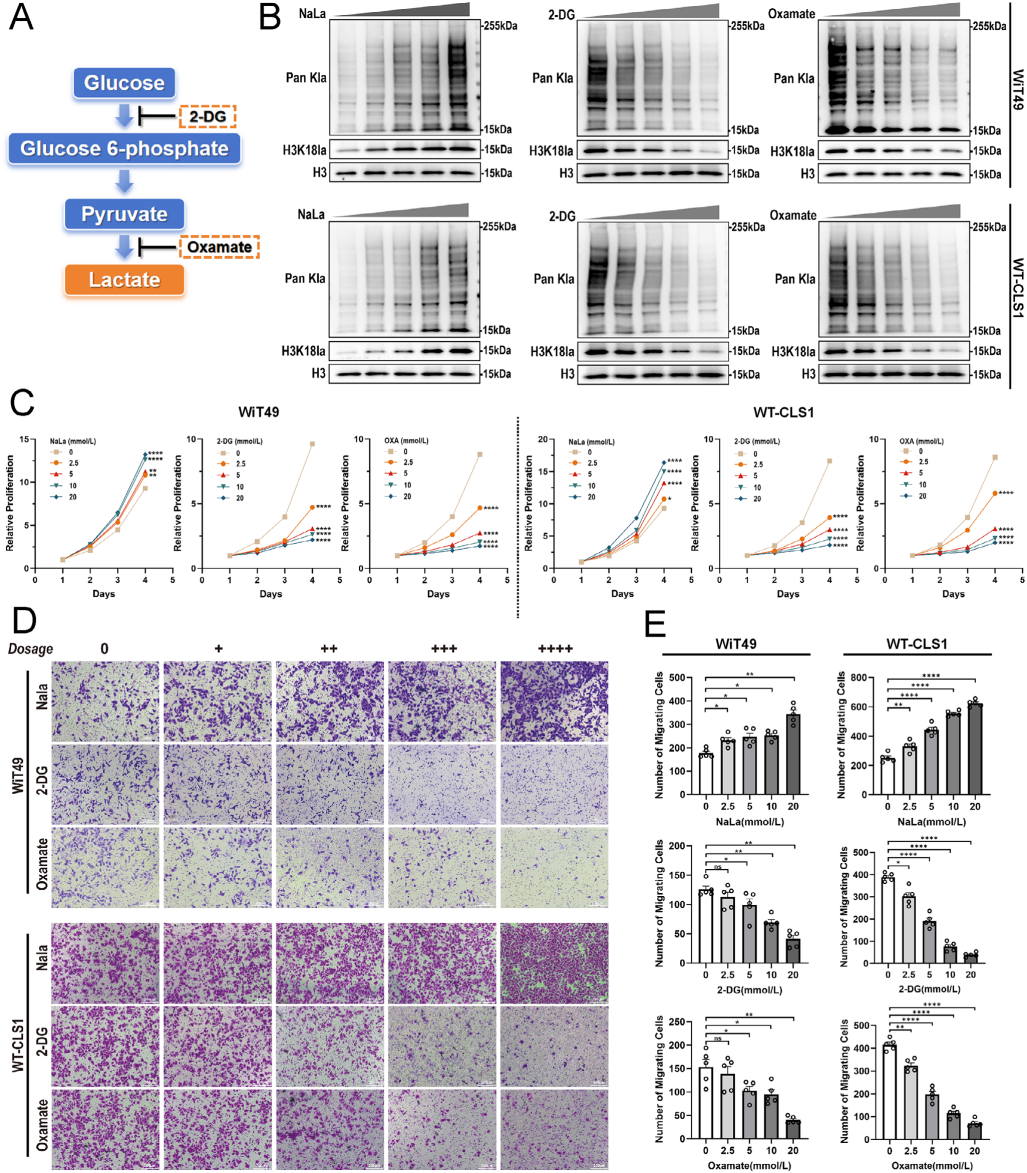
Modulation of Histone Lactylation Levels Affects WT Cell Proliferation and Migration. **(A)** Schematic diagram of targets for inhibiting histone lactylation. (**B)** WB images of Pan Kla and H3K18la levels in WiT49 and WT‐CLS1 cells treated with gradient doses of NaLa, 2‐DG, or oxamate (0, 2.5, 5, 10, 20 mM) (*n* = 3). (**C)** CCK‐8 assay results showing the proliferation of WiT49 and WT‐CLS1 cells after treatment with NaLa, 2‐DG, or oxamate (*n* = 3). (**D, E)** Transwell assay images (D) and quantitative analysis of migration capabilities (E) of WiT49 and WT‐CLS1 cells after the above treatments (*n* = 5). Data are presented as mean ± SD. ^*^ *p* < 0.05, ^**^ *p* < 0.01, ^***^ *p* < 0.001, ^****^ *p* < 0.0001.

To further elucidate the role of histone lactylation in malignant tumor progression at the cellular level, we investigated how modulating histone lactylation levels affects the proliferation and migration of WT cell lines. Prior studies have shown that supplementation with exogenous sodium lactate (NaLa) can enhance histone lactylation levels, whereas 2‐deoxy‐D‐glucose (2‐DG, a non‐metabolizable glucose analog) and oxamate are well‐established glycolysis inhibitors that can reduce lactate production (Figure 2A). We therefore utilized these three agents to manipulate intracellular lactate levels, thereby upregulating or downregulating histone lactylation. After optimizing drug concentrations through preliminary experiments, WB assays confirmed that Pan Kla and H3K18la levels in both WiT49 and WT‐CLS1 cell lines were altered in a dose‐dependent manner following drug treatment (Figure 2B; Figure S1B). Consistently, CCK‐8 proliferation assays (Figure 2C) and Transwell migration assays (Figure 2D,E) conducted in both cell lines yielded congruent results: NaLa treatment significantly enhanced the proliferation and migration capacities of WT cells, whereas administration of 2‐DG or oxamate led to a marked reduction in these malignant phenotypes.

In conclusion, our preliminary findings indicate that aberrantly activated glycolysis in Wilms tumor drives intracellular lactate accumulation, which in turn enhances histone lactylation, particularly H3K18la modification, and consequently promotes the proliferation and migration of tumor cells.

### 3.2 H3K18la Drives Transcription of PSRC1, a Key Downstream Target Gene Correlated With Prognosis in WT

**FIGURE 3:**
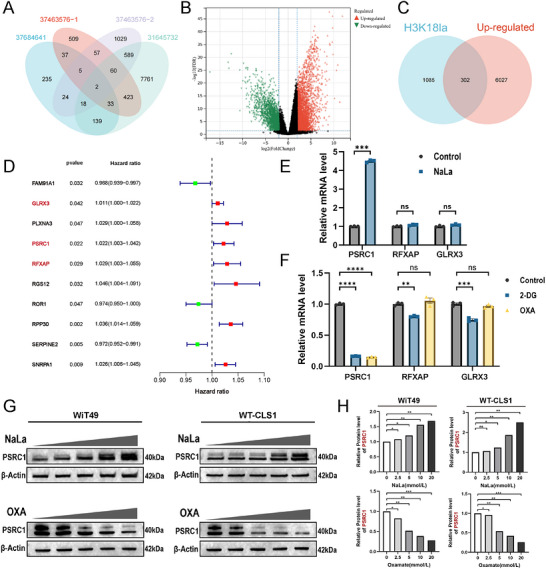
Screening and Validation of PSRC1 as a Key Downstream Target of H3K18la. (**A)** Venn diagram showing 1,387 candidate genes bound by H3K18la antibody in at least two ChIP‐seq datasets (PMID: 37463576; 37684641; 31645732). (**B)** Volcano plot of differentially expressed genes (|log2FC| > 2, FDR < 0.05) between WT and normal groups in the TARGET database. (**C)** Venn diagram showing 302 overlapping genes between predicted H3K18la‐bound genes (*n* = 1387) and differentially upregulated genes (*n* = 6329). (**D)** Forest plot of 10 genes significantly associated with survival identified by univariate Cox regression analysis (*n* = 126; *p* < 0.05). (**E, F)** qPCR analysis of mRNA expression levels of PSRC1, RFXAP, and GLRX3 in WiT49 cells after treatment with NaLa (E), 2‐DG, or oxamate (F). (**G, H)** WB images (G) and quantitative results (H) of PSRC1 protein levels in WiT49 and WT‐CLS1 cells treated with NaLa or oxamate. ^*^ *p* < 0.05, ^**^ *p* < 0.01, ^***^ *p* < 0.001, ^****^ *p* < 0.0001.

To identify potential downstream target genes of H3K18la, we integrated H3K18la public ChIP‐seq datasets [16, 26, 27] with RNA‐seq datasets of WT from the TARGET database. To mitigate the risk of false positives and false negatives inherent to ChIP‐seq data, we screened for target genes that were bound by the H3K18la antibody in at least two independent sequencing datasets, yielding a total of 1,387 candidate genes (Figure 3A). Subsequently, we performed differential expression analysis on the TARGET database to identify genes with significantly upregulated expression in the WT group relative to the normal group (|log_2_FC| > 2; FDR < 0.05). This analysis identified 6,329 differentially upregulated candidate genes (Figure 3B). The intersection of these two candidate gene sets yielded 302 overlapping genes (Figure 3C). To further narrow down the candidate pool to clinically relevant genes, we retrieved clinical follow‐up data for 126 WT patients from the TARGET database and subjected the 302 overlapping genes to univariate and multivariate Cox proportional hazards regression analyses. Univariate Cox regression analysis initially identified 10 genes with a significant correlation with patient survival (*p* < 0.05) (Figure 3D), which were subsequently included in the multivariate regression analysis (Figure S2A). After excluding protective factors, we ultimately identified three genes with independent prognostic significance: PSRC1, RFXAP, and GLRX3.

To further identify the target genes that act as the key downstream targets of histone lactylation in WT, we used qPCR to assess changes in the mRNA expression levels of these genes in WiT49 cells after treatment with NaLa, 2‐DG, or oxamate (Figure 3E,F). The results showed that only PSRC1 exhibited a distinct expression pattern: its mRNA levels were significantly elevated by NaLa treatment and markedly reduced by 2‐DG or oxamate administration. Intriguingly, the other two genes also showed a noticeable decrease in mRNA levels following 2‐DG treatment. This observation may be attributed to the fact that 2‐DG, as a broad‐spectrum glycolysis inhibitor, not only suppresses lactate production but also induces global cellular energy stress and signaling pathway disruption, which may non‐specifically impair the transcription of multiple genes. Next, we performed WB assays to examine PSRC1 protein expression in two WT cell lines (WiT49 and WT‐CLS1) treated with NaLa or oxamate. The results further confirmed that PSRC1 expression in WT cells is directly modulated by intracellular lactylation levels (Figure 3G,H). Collectively, these findings indicated that PSRC1 expression exhibited a stronger and more specific correlation with lactylation levels, prompting us to focus on PSRC1 for subsequent investigations.

**FIGURE 4:**
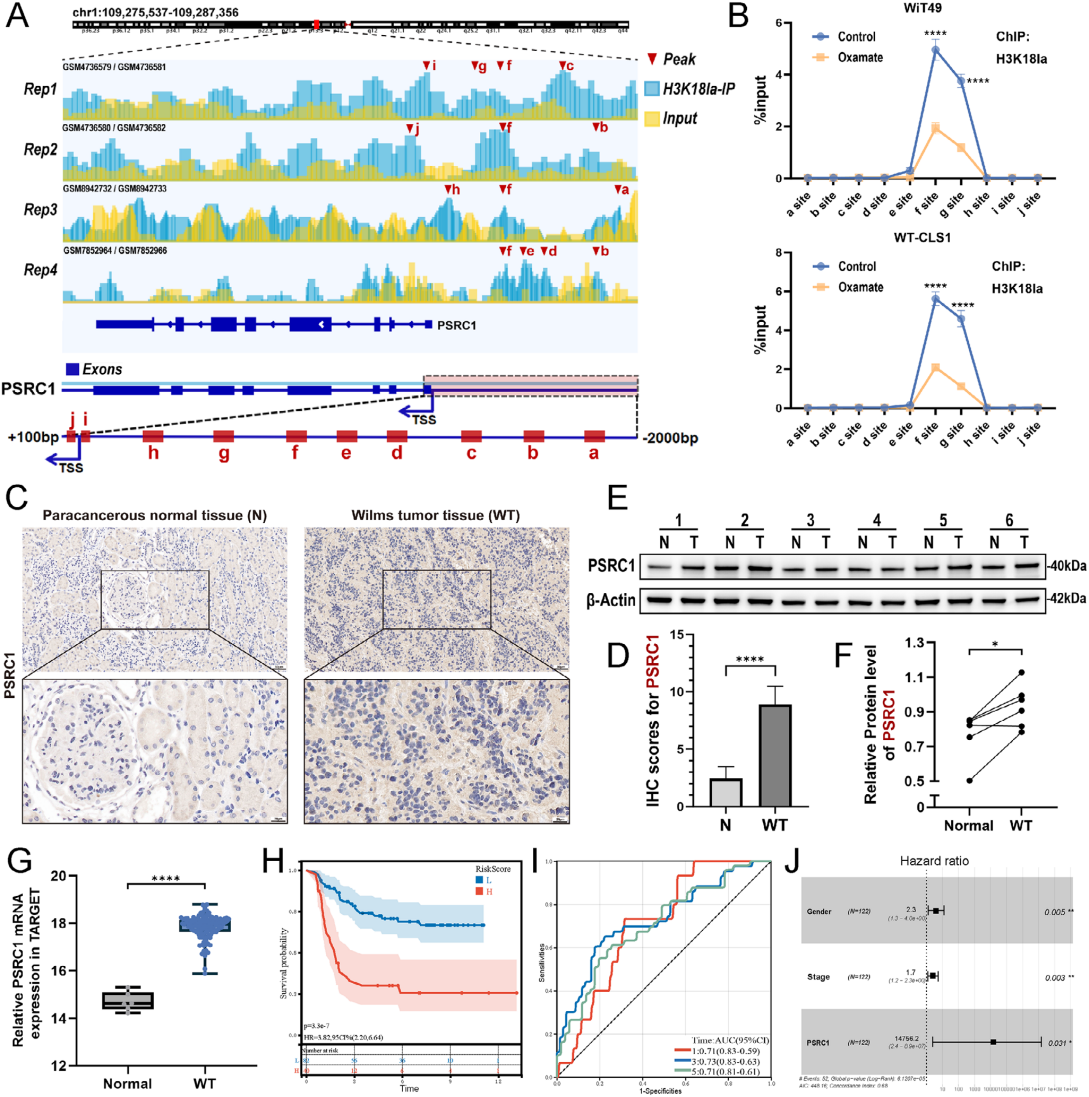
PSRC1 Is Directly Regulated by H3K18la and Correlates With WT Prognosis. (**A)** Schematic diagram of 10 potential H3K18la binding sites in the PSRC1 promoter region based on ChIP‐seq data (GSE156675; GSE295142; GSE245990). (**B)** ChIP‐qPCR results showing relative H3K18la enrichment in the PSRC1 promoter region in WiT49 and WT‐CLS1 cells with or without oxamate treatment. (**C, D)** Representative IHC images (C) and IHC scores (D) of PSRC1 protein expression in WT tissues and adjacent normal (N) tissues (*n* = 52). (**E, F)**. WB images (E) and quantitative results (F) of PSRC1 protein levels in WT tissues and adjacent normal (N) tissues (*n* = 6). (**G)** Differential expression analysis of PSRC1 mRNA levels between WT and normal groups in the TARGET database. (**H)** K‐M survival curves of WT patients stratified by PSRC1 expression levels (*n* = 122). (**I)** ROC curves showing the predictive accuracy of PSRC1 for 1‐year, 3‐year, and 5‐year survival (AUC = 0.71, 0.73, 0.71). Data are presented as mean ± SD. ^*^ *p* < 0.05, ^**^ *p* < 0.01, ^***^ *p* < 0.001, ^****^ *p* < 0.0001.

To further verify that PSRC1 transcription is directly activated by H3K18la modification, we analyzed ChIP‐seq datasets (GSE156675; GSE295142; GSE245990) and identified ten potential H3K18la binding sites within the PSRC1 promoter region, for which we designed specific primers (Figure 4A). ChIP‐qPCR assays revealed robust enrichment of H3K18la at the PSRC1 promoter region in both WT cell lines, and this enrichment was significantly reduced by treatment with oxamate (Figure 4B). At the tissue level, we conducted IHC (Figure 4C,D) and WB (Figure 4E,F) assays to evaluate PSRC1 expression in paired WT tissues and adjacent normal tissues. The results demonstrated that PSRC1 protein expression was significantly higher in WT tissues than in adjacent normal tissues. To validate these findings in an independent cohort, we performed systematic bioinformatic analyses using the TARGET database: differential expression analysis confirmed that PSRC1 mRNA levels were significantly upregulated in WT tissues relative to normal tissues (Figure 4G); K‐M survival analysis showed that patients in the high‐PSRC1 expression group had significantly poorer overall survival (OS) than those in the low‐PSRC1 expression group (Figure 4H); receiver operating characteristic (ROC) curve analysis yielded area under the curve (AUC) values of 0.71, 0.73, and 0.71 for 1‐year, 3‐year, and 5‐year survival predictions, respectively, indicating moderate predictive accuracy (Figure 4I). Multivariate Cox regression analysis incorporating multiple clinical variables (age, gender, clinical stage, and PSRC1 expression level) demonstrated that high PSRC1 expression is an independent prognostic risk factor (Figure 4J). To facilitate clinical application, we constructed a nomogram to visually depict the association between three independent prognostic factors (gender, clinical stage, and PSRC1 expression) and the probability of OS at 1, 3, and 5 years (Figure S2B).

### 3.3 p300 Regulates PSRC1 Specifically via H3K18 Lactylation

**FIGURE 5:**
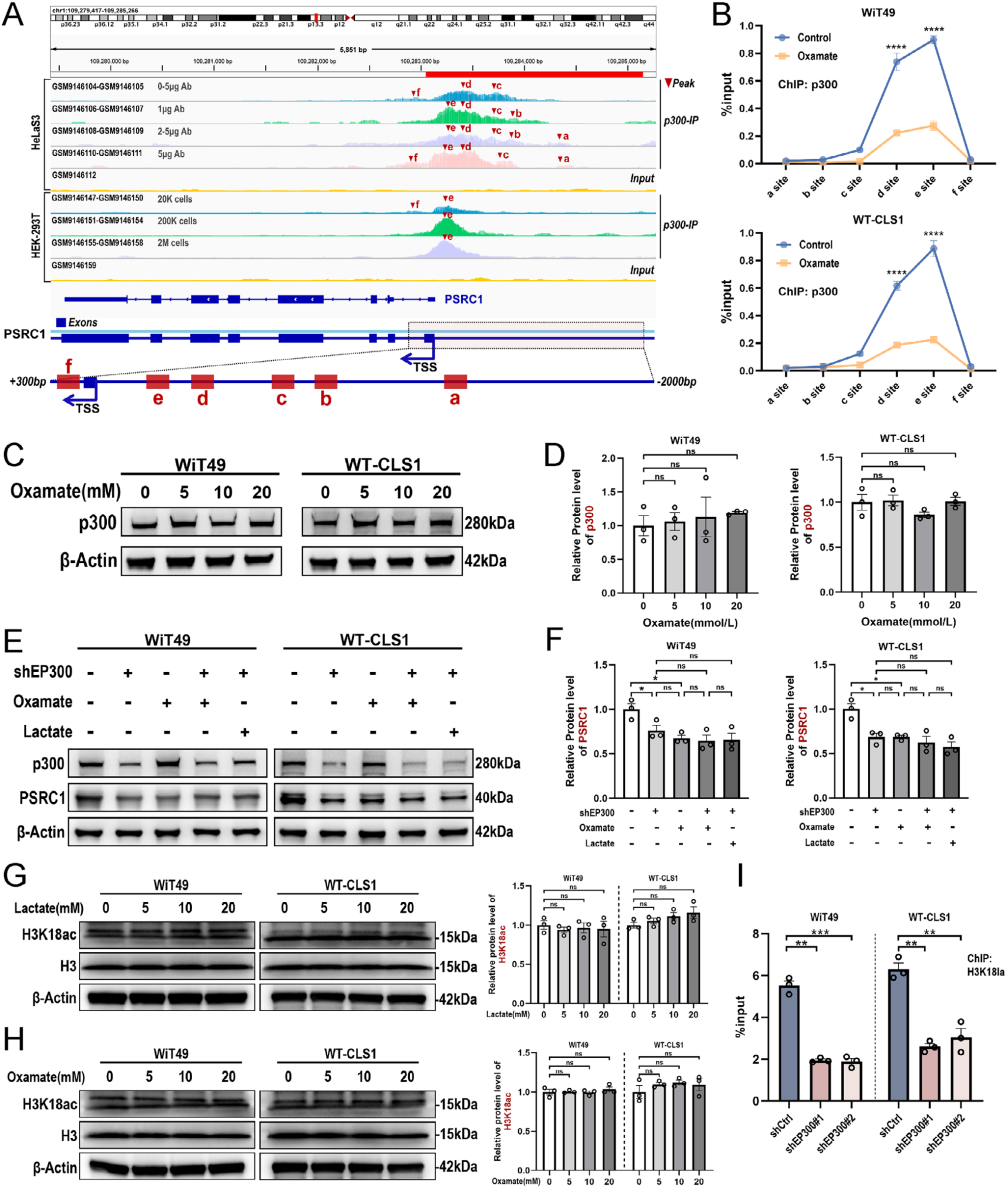
P300 Regulates PSRC1 Specifically via H3K18 Lactylation. (**A)** Schematic diagram of 6 potential p300 binding sites in the PSRC1 promoter region based on ChIP‐seq data (GSE304259). (**B)** ChIP‐qPCR results showing relative p300 enrichment in the PSRC1 promoter region in WiT49 and WT‐CLS1 cells with or without oxamate treatment. (**C, D)** WB images (C) and quantitative analysis (D) of relative p300 protein expression in WiT49 and WT‐CLS1 cells treated with oxamate (*n* = 3). (**E, F)** WB images (E) and quantitative analysis (F) of relative PSRC1 protein levels in WiT49 and WT‐CLS1 cells after p300 knockdown, oxamate treatment, combined p300 knockdown and oxamate treatment, or p300 knockdown plus lactate supplementation (*n* = 3). (**G, H)** WB of H3K18ac in WiT49 and WT‐CLS1 cells treated with gradient NaLa (G) or oxamate (H) (Total H3 as loading control; *n* = 3). (**I)** ChIP‐qPCR of H3K18la enrichment at the PSRC1 promoter in WT cells transduced with shCtrl, shEP300#1 or shEP300#2 (*n* = 3). Data are presented as mean ± SD. ^*^ *p* < 0.05, ^**^ *p* < 0.01, ^***^ *p* < 0.001, ^****^ *p* < 0.0001.

Notably, accumulating evidence has demonstrated that p300 functions as a histone lactylation “writer” in multiple cancer types [17], but this role has not yet been validated in Wilms tumor. To address this gap, we analyzed ChIP‐seq data (GSE304259) and identified six potential p300 binding sites within the PSRC1 promoter region, for which we designed specific primers (Figure 5A). ChIP‐qPCR assays showed that p300 was enriched at the PSRC1 promoter region in both WT cell lines, and this enrichment was significantly reduced following oxamate treatment (Figure 5B). Western blot analysis confirmed that oxamate treatment did not alter total p300 protein expression levels in WT cell lines (Figure 5C,D). This finding, combined with our observation that oxamate diminishes H3K18la deposition at the PSRC1 promoter region (Figure 4B), indicates that oxamate impairs p300 binding and its subsequent lactylation activity at the PSRC1 locus by depleting the essential substrate lactate, rather than by reducing the abundance of the p300 enzyme itself. Moreover, shRNA‐mediated knockdown of p300 led to a significant decrease in PSRC1 protein levels (Figure 5E,F; Figure S3A). Notably, in cells with p300 knockdown, neither oxamate treatment nor exogenous lactate supplementation was able to alter PSRC1 expression (Figure 5E,F). This confirms that p300 is indispensable for translating the lactate signal into PSRC1 transcription.

Given that p300 is also a well‐established histone acetyltransferase, we sought to ascertain whether its regulation of PSRC1 is mediated specifically through lactylation rather than acetylation. To address this, we first assessed whether alterations in intracellular lactate levels could inadvertently affect histone acetylation at the same residue. Western blot analysis revealed that while H3K18la levels exhibited dose‐dependent changes in response to both sodium lactate (NaLa) and oxamate treatment (as previously shown in Figure 2B), H3K18ac (Histone H3 lysine 18 acetylation) protein levels remained unchanged across all tested concentrations in both WiT49 and WT‐CLS1 cells (Figure 5G,H). We next performed ChIP‐qPCR to directly examine whether p300 is required for H3K18la deposition at the PSRC1 promoter. Based on our initial mapping of H3K18la binding sites (Figure 4B), we selected “f site” as a representative locus for further analysis. In both WT cell lines, transduction with two independent shRNAs targeting p300 (shEP300#1 and shEP300#2) significantly reduced H3K18la enrichment at the PSRC1 promoter region compared to control groups (Figure 5I).

Taken together, these lines of evidence strongly suggest that PSRC1 acts as a key downstream effector of H3K18la in WT. Its transcription is tightly regulated by both intracellular lactylation levels and the histone lactylation writer p300, which mediates the conversion of lactate‐driven metabolic signals into a pro‐tumorigenic transcriptional program, thereby promoting the malignant progression of WT.

### 3.4 The H3K18la/PSRC1 Axis Activates the AKT/mTOR/HIF‐1α Signaling Pathway

**FIGURE 6:**
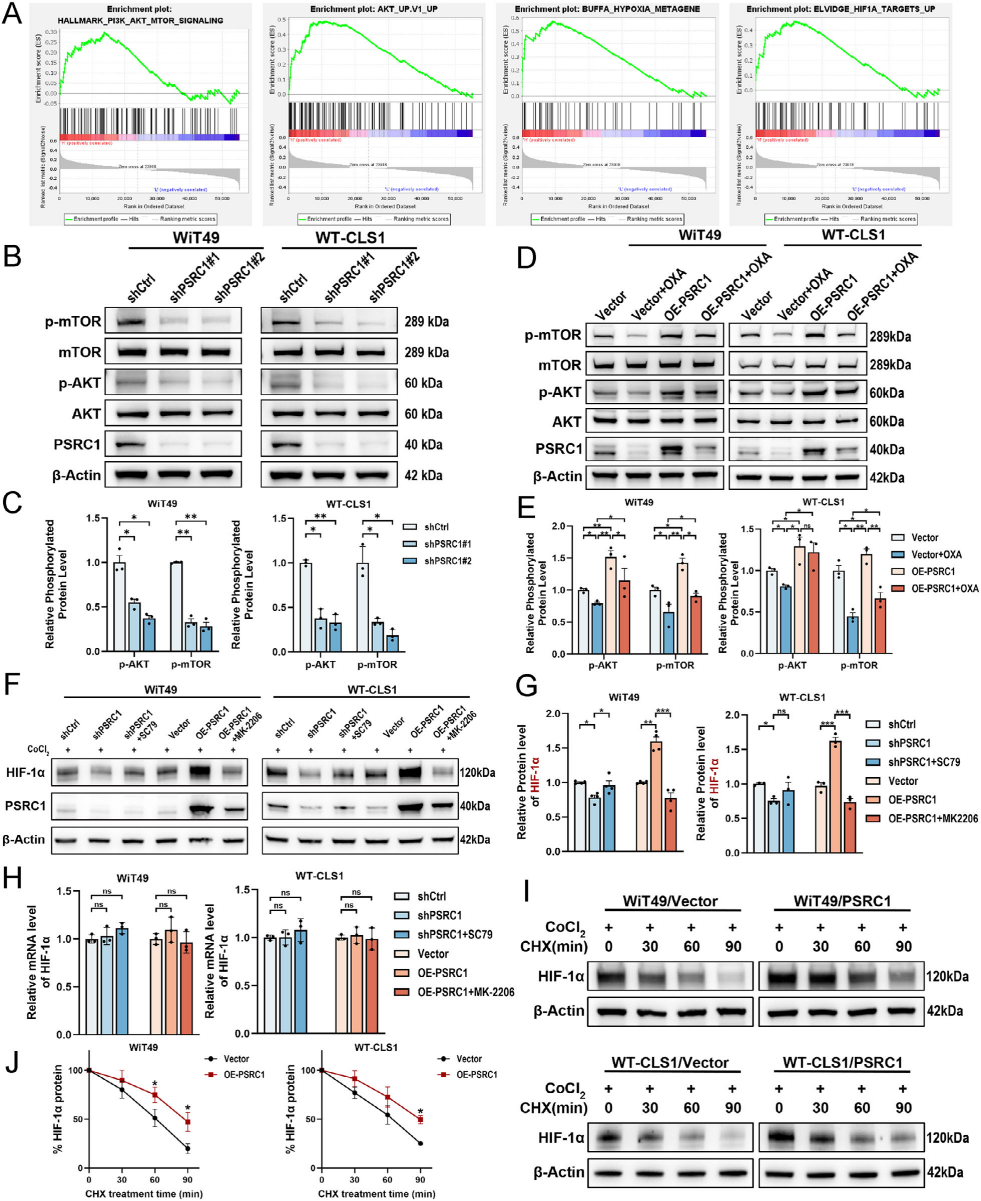
PSRC1 Activates the AKT/mTOR/HIF‐1α Signaling Pathway. (**A)** GSEA analysis showing that AKT/mTOR and HIF‐1α‐related signaling pathways are significantly enriched in the high‐PSRC1 expression group (*p* < 0.05). (**B, C)** WB images (B) and quantitative analysis of relative p‐AKT and p‐mTOR protein levels (C) in WiT49 and WT‐CLS1 cells (shCtrl, shPSRC1#1, shPSRC1#2 groups; *n* = 3 per group). (**D, E)** WB images (D) and quantitative analysis of relative p‐AKT and p‐mTOR protein levels (E) in WiT49 and WT‐CLS1 cells (Vector, Vector+OXA, OE‐PSRC1, OE‐PSRC1+OXA groups; *n* = 3 per group). (**F, G)** WB images (F) and quantitative analysis of relative HIF‐1α protein levels (G) in WiT49 and WT‐CLS1 cells (shCtrl, shPSRC1, shPSRC1+SC79, Vector, OE‐PSRC1, OE‐PSRC1+MK2206 groups; *n* = 3 per group). (**H)** Relative mRNA levels of HIF‐1α by qPCR in WiT49 and WT‐CLS1 cells (shCtrl, shPSRC1, shPSRC1+SC79, Vector, OE‐PSRC1, OE‐PSRC1+MK2206 groups; *n* = 3 per group). (**I, J)** HIF‐1α degradation rate was determined by treating hypoxic WiT49 and WT‐CLS1 cells (control vs. OE‐PSRC1) with cycloheximide (CHX, 100 µg/mL). Data are presented as mean ± SD. ^*^ *p* < 0.05, ^**^ *p* < 0.01, ^***^ *p* < 0.001, ^****^ *p* < 0.0001.

Having established that PSRC1 acts as a critical pro‐tumorigenic effector downstream of H3K18la, we next sought to explore the underlying molecular mechanisms. GSEA revealed that the AKT/mTOR and HIF‐1α signaling pathways were significantly enriched in the high‐PSRC1 expression group (Figure 6A). Given that prior studies have demonstrated that the AKT/mTOR pathway can transcriptionally upregulate HIF‐1α protein expression [28], we hypothesized that the H3K18la/PSRC1 axis might promote WT progression by activating the AKT/mTOR/HIF‐1α signaling cascade.

WB assays showed that both PSRC1 knockdown and oxamate treatment significantly inhibited the phosphorylation of AKT and mTOR in WT cells (Figure 6B–E; Figure S3B,C). Conversely, PSRC1 overexpression enhanced the phosphorylation of AKT and mTOR and partially reversed the pathway inhibition induced by oxamate (Figure 6D,E). To further explore the regulatory link between the AKT/mTOR pathway and HIF‐1α, we treated WT cells with cobalt chloride (CoCl_2_, a hypoxia‐mimicking agent) at a consistent concentration. The results showed that PSRC1 knockdown decreased HIF‐1α protein levels, whereas PSRC1 overexpression increased HIF‐1α expression (Figure 6F,G; Figure S3D). Moreover, treatment with the AKT activator SC79 effectively reversed the reduction in HIF‐1α levels caused by PSRC1 knockdown, while the AKT inhibitor MK2206 abrogated the increase in HIF‐1α expression induced by PSRC1 overexpression (Figure 6F,G). Intriguingly, neither PSRC1 knockdown nor overexpression altered the mRNA expression level of HIF‐1α (Figure 6H), implying that PSRC1 regulates HIF‐1α expression at the post‐transcriptional level. To confirm this, we performed protein half‐life assays, which demonstrated that PSRC1 overexpression significantly prolonged the half‐life of HIF‐1α protein in WT cells (Figure 6I,J).

Collectively, these findings indicate that PSRC1 enhances HIF‐1α protein stability at the post‐transcriptional level by activating the AKT/mTOR signaling pathway, thus uncovering a key molecular mechanism mediating the pro‐tumorigenic effects of histone lactylation in Wilms tumor.

### 3.5 PSRC1 Promotes WT Progression and Partially Reverses the Tumor‐Suppressive Effect of Oxamate In Vitro and In Vivo

**FIGURE 7:**
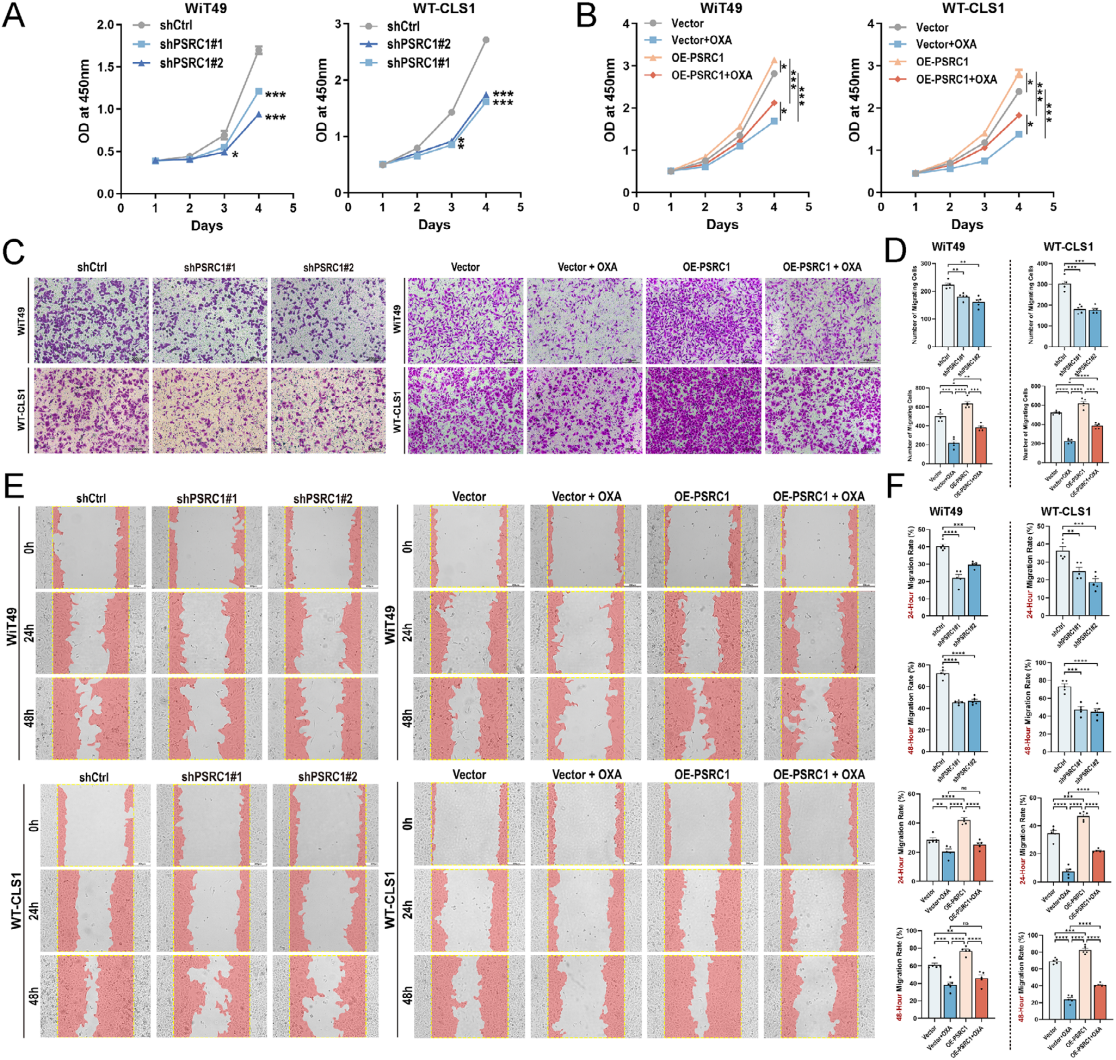
PSRC1 Promotes WT Progression and Partially Reverses the Tumor‐Suppressive Effect of Oxamate *in Vitro. (* **A)** CCK‐8 assay results of WiT49 and WT‐CLS1 cells in shCtrl and shPSRC1 groups (*n* = 3 per group). (**B)** CCK‐8 assay results of WiT49 and WT‐CLS1 cells in Vector, Vector+Oxamate (OXA), OE‐PSRC1, and OE‐PSRC1+OXA groups (*n* = 3). (**C, D)** Transwell assay results (C) and quantitative analysis (D) of WiT49 and WT‐CLS1 cells in the above groups (*n* = 5). (**E, F)** Wound healing assay results (E) and quantitative analysis (F) of WiT49 and WT‐CLS1 cells in the above groups (*n* = 5). Data are presented as mean ± SD. ^*^ *p* < 0.05, ^**^ *p* < 0.01, ^***^ *p* < 0.001, ^****^ *p* < 0.0001.

To validate the pro‐tumorigenic roles of histone lactylation and PSRC1 in Wilms tumor, we first assessed the impact of PSRC1 expression on the proliferation and migration of two WT cell lines (WiT49 and WT‐CLS1) via in vitro assays. CCK‐8 proliferation assays showed that PSRC1 knockdown significantly suppressed the proliferative capacity of WT cells (Figure 7A). In contrast, PSRC1 overexpression not only enhanced tumor cell proliferation but also partially abrogated the growth‐inhibitory effect of oxamate (Figure 7B). Consistently, Transwell migration (Figure 7C,D) and wound healing (Figure 7E,F) assays demonstrated that PSRC1 knockdown impaired the migratory potential of WT cells, whereas PSRC1 overexpression promoted cell migration and partially reversed the migration‐suppressive effect induced by oxamate treatment.

**FIGURE 8:**
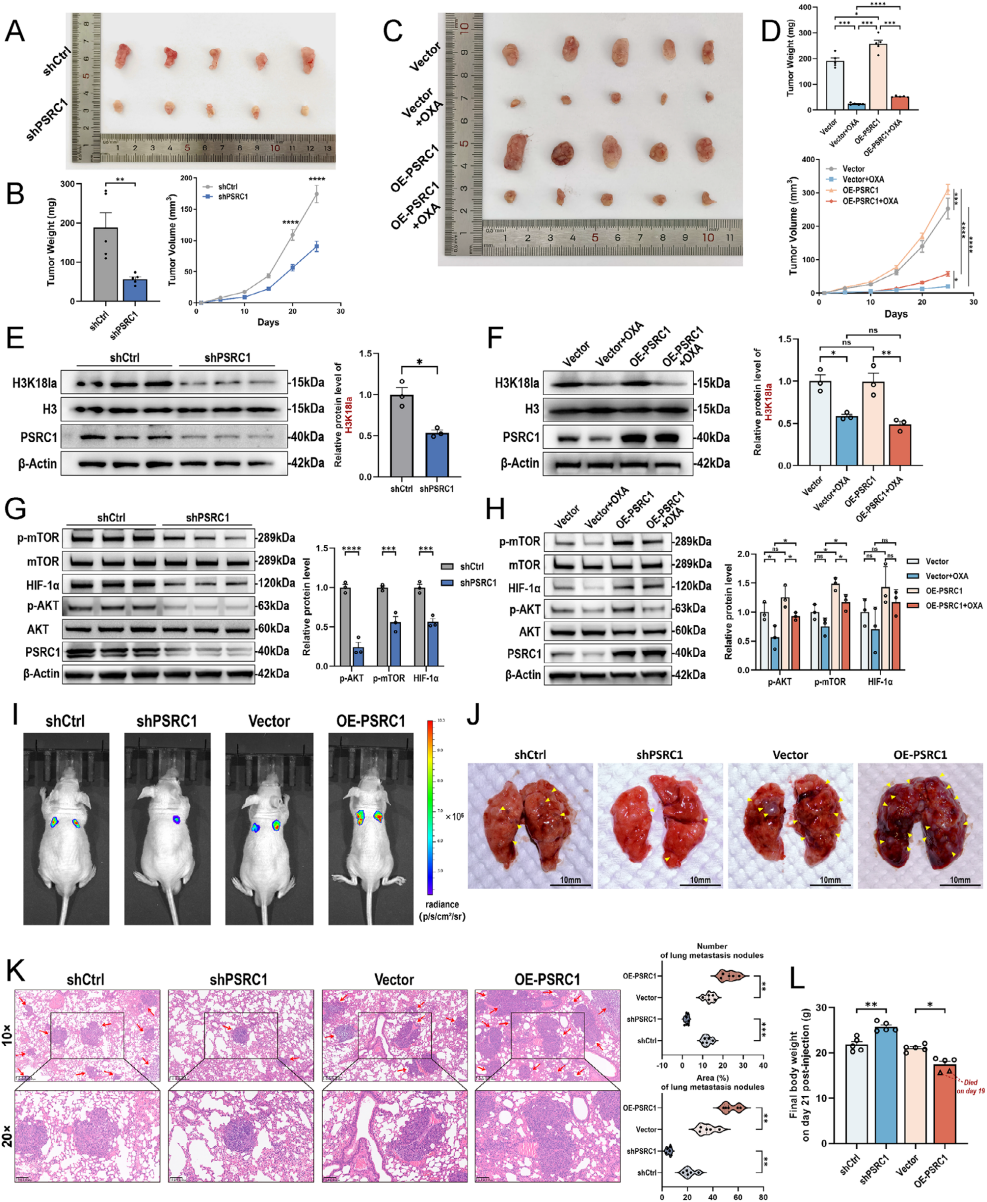
PSRC1 Promotes Wilms Tumor Growth and Metastasis *in Vivo. (* **A, B)** Subcutaneous xenograft tumor images (A) and tumor growth curves with final tumor volumes (B) from BALB/c nude mice in shCtrl and shPSRC1 groups (*n* = 5). (**C, D)** Subcutaneous xenograft tumor images (C) and tumor growth curves with final tumor volumes (D) from BALB/c nude mice in Vector, Vector + Oxamate (OXA), OE‐PSRC1, and OE‐PSRC1 + OXA groups (*n* = 5). (**E–H)** WB of H3K18la, H3, PSRC1, p‐AKT, AKT, p‐mTOR, mTOR, HIF‐1α and β‐Actin in xenograft tumors (*n* = 3). (**I)** Representative bioluminescence images of lung metastases in mice injected with shCtrl, shPSRC1, Vector, or OE‐PSRC1 WiT49 cells. (**J)** Representative gross lung images. **(K)** HE‐stained sections and quantification of metastatic nodules (*n* = 5). (**L)** Final body weights at day 21 post‐injection of mice (*n* = 5). Data are presented as mean ± SD. ^*^ *p* < 0.05, ^**^ *p* < 0.01, ^***^ *p* < 0.001, ^****^ *p* < 0.0001.

To further corroborate these findings in vivo, we established BALB/c nude mouse xenograft models of WT. WiT49 cells pre‐treated with oxamate and/or PSRC1 overexpression plasmids were subcutaneously inoculated into BALB/c nude mice. Mice received daily oxamate administration (i.p., 500 mg/kg) according to the assigned experimental groups. Tumor volumes were measured with a vernier caliper every 5 days until the mice were euthanized on day 25. As anticipated, the tumor growth rate and final volume in the PSRC1‐knockdown group were markedly reduced compared with the control group (Figure 8A,B; Figure S4A). Similarly, oxamate treatment led to significant tumor growth inhibition (Figure 8C,D; Figure S4B). Meanwhile, PSRC1 overexpression not only accelerated the growth of subcutaneous xenograft tumors but also partially reversed the tumor‐suppressive effect of oxamate (Figure 8C,D). Western blot analysis of xenograft tumor tissues confirmed that the signaling axis established in vitro is recapitulated in vivo. In PSRC1‐knockdown tumors, H3K18la levels were markedly lower than in controls (Figure 8E; Figure S4C), likely reflecting both reduced tumor hypoxia (due to a smaller, better‐perfused tumor mass) and a potential positive feedback mechanism between H3K18la and PSRC1. Oxamate suppressed H3K18la regardless of PSRC1 overexpression, and OE‐PSRC1 failed to reverse this reduction (Figure 8F; Figure S4D), confirming that oxamate acts upstream through substrate depletion. Downstream, PSRC1 knockdown significantly decreased p‐AKT, p‐mTOR, and HIF‐1α levels (Figure 8G; Figure S4E), providing in vivo evidence that PSRC1 sustains the AKT/mTOR pathway and HIF‐1α stability. In the oxamate/OE‐PSRC1 groups (Figure 8H; Figure S4F), p‐AKT and p‐mTOR trends recapitulated in vitro findings: oxamate reduced phosphorylation, while OE‐PSRC1 enhanced it and partially rescued oxamate‐induced suppression. HIF‐1α showed similar trends but with greater intra‐group variability and no statistical significance, likely due to the dominant influence of regional intratumoral hypoxia over pharmacological or genetic manipulations, as well as random sampling of tumor regions during tissue processing. Collectively, these in vivo data corroborate the H3K18la/PSRC1/AKT/mTOR/HIF‐1α regulatory axis.

We further evaluated the prometastatic role of PSRC1 using a tail vein injection lung metastasis model. Bioluminescence imaging showed that PSRC1 knockdown significantly reduced pulmonary metastatic burden relative to shCtrl, whereas PSRC1 overexpression enhanced lung colonization compared to Vector (Figure 8I). Gross examination and HE staining of lung sections confirmed fewer and smaller metastatic nodules in the shPSRC1 group, with more extensive tumor infiltration in OE‐PSRC1 lungs (Figure 8J,K). Mice in the shPSRC1 group maintained relatively higher body weights than controls, whereas OE‐PSRC1 mice were generally lean with lower body weights, and two animals in this group required euthanasia on day 19 due to cachexia (Figure 8L). These in vivo findings corroborate our in vitro migration data and establish PSRC1 as a promoter of Wilms tumor metastasis.

### 3.6 PSRC1 Competes With PTEN for AKT Binding to Promote AKT Phosphorylation

**FIGURE 9:**
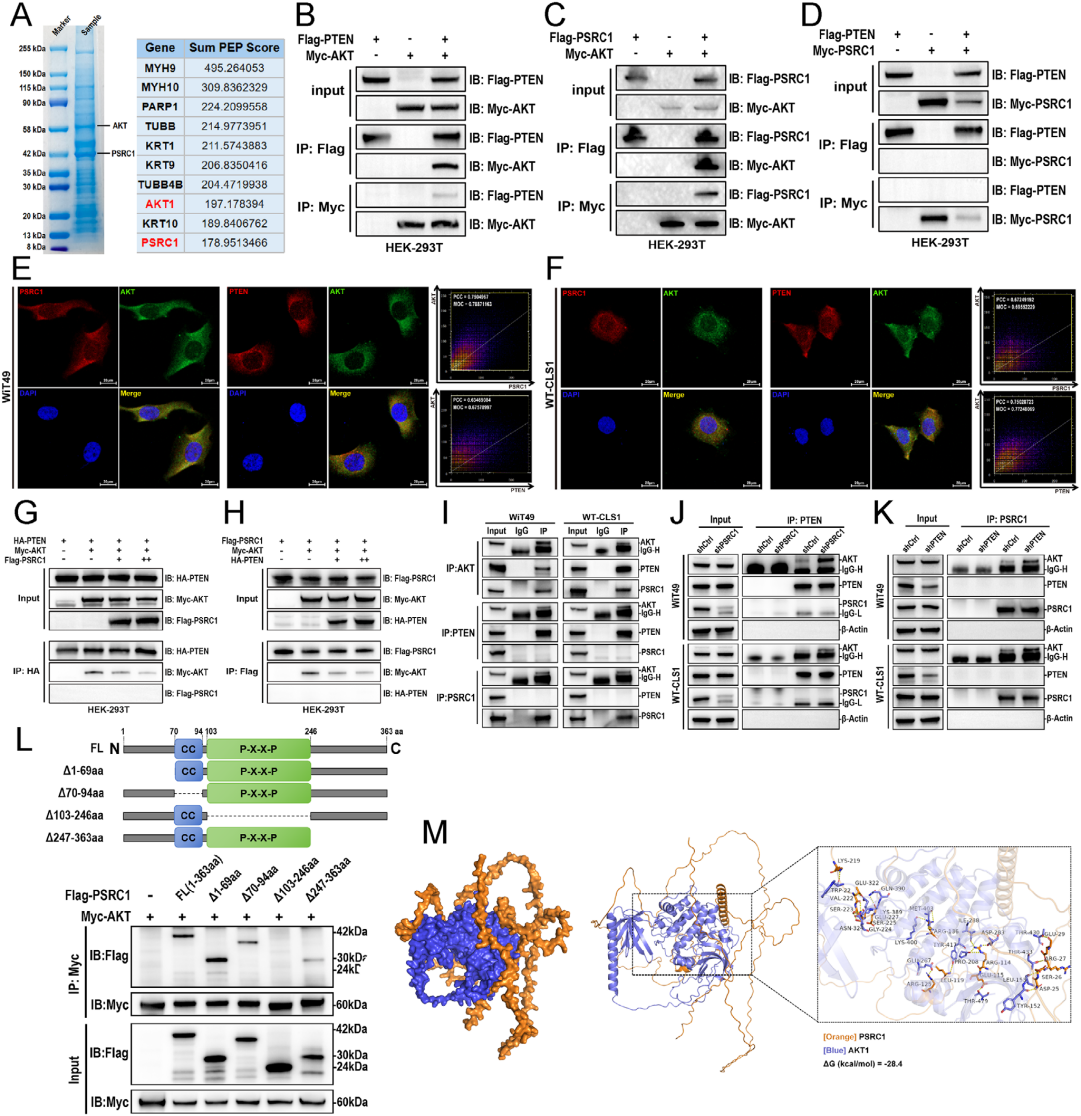
PSRC1 Competes With PTEN for AKT Binding. (**A)** Mass spectrometry (MS) results showing potential PSRC1‐interacting proteins. (**B–D)** Exogenous Co‐IP assay results showing the interaction between PTEN and AKT (B), PSRC1 and AKT (C), and the absence of interaction between PSRC1 and PTEN (D) in HEK‐293T cells. (**E, F)** Immunofluorescence (IF) images showing the co‐localization of PSRC1/PTEN with AKT in WiT49 (E) and WT‐CLS1 (F) cells. (**G, H)** Exogenous Co‐IP assay results showing the effect of PSRC1 overexpression on PTEN‐AKT binding (G) and the effect of PTEN overexpression on PSRC1‐AKT binding (H) in HEK‐293T cells. (**I)** Endogenous Co‐IP assay results showing the interaction among PSRC1, PTEN, and AKT in WiT49 and WT‐CLS1 cells. (**J, K)** Endogenous Co‐IP assay results showing the effect of KD‐PSRC1 on PTEN‐AKT binding (J) and the effect of KD‐PTEN on PSRC1‐AKT binding (K) in WiT49 and WT‐CLS1 cells. (**L)** Schematic diagram of truncation constructs for PSRC1 and Co‐IP assay results identifying the key domain for PSRC1‐AKT interaction. (**M)** Molecular docking results showing the specific binding sites and binding energy (ΔG = −28.4 kcal/mol) between PSRC1 and AKT1.

To elucidate the precise mechanism by which PSRC1 regulates the AKT/mTOR/HIF‐1α pathway, we first predicted PSRC1‐interacting proteins using the BioGRID database (Figure S5A) and validated these interactions via mass spectrometry (MS) (Figure 9A; Figure S5B,C). Notably, AKT was identified as a potential binding partner of PSRC1. PTEN is a well‐characterized lipid phosphatase that indirectly inhibits AKT activation. Additionally, it exerts protein phosphatase activity by directly binding to AKT, thereby mediating its dephosphorylation and inactivation [29]. Notably, a competitive binding model between mitosis‐related proteins and PTEN for AKT has been previously reported in other malignancies [30, 31], providing a precedent for investigating whether mitosis‐related PSRC1 may function through an analogous mechanism. To clarify the binding relationships among PSRC1, PTEN, and AKT, we performed exogenous co‐immunoprecipitation (Co‐IP) assays in HEK‐293T cells. The results showed that both PTEN and PSRC1 could bind to AKT (Figure 9B,C), whereas no direct interaction was detected between PTEN and PSRC1 (Figure 9D). Immunofluorescence (IF) staining further confirmed that both PTEN and PSRC1 co‐localized with AKT in the cytoplasm and nucleus of WiT49 and WT‐CLS1 cells (Figure 9E,F).

Based on these observations, we hypothesized that PSRC1 promotes AKT phosphorylation by competing with PTEN for AKT binding, thereby activating downstream pro‐tumorigenic signaling pathways. To test this hypothesis, we conducted additional exogenous Co‐IP assays in HEK‐293T cells. The results showed that PSRC1 overexpression diminished the interaction between AKT and PTEN (Figure 9G), while PTEN overexpression diminished the interaction between AKT and PSRC1 (Figure 9H). To validate these findings in a more physiologically relevant context, we performed endogenous Co‐IP assays in WiT49 and WT‐CLS1 cells. Consistent with the results from HEK‐293T cells, PSRC1, PTEN, and AKT exhibited the same binding patterns in WT cell lines (Figure 9I). Furthermore, in WT cells, PSRC1 knockdown significantly enhanced the PTEN‐AKT interaction (Figure 9J), whereas PTEN knockdown strengthened the binding between PSRC1 and AKT (Figure 9K). Taken together, these results strongly support the conclusion that PSRC1 and PTEN competitively bind to AKT.

To identify the specific domain of PSRC1 responsible for AKT binding, we retrieved the amino acid sequence of PSRC1 from the UniProt database: full‐length PSRC1 comprises 363 amino acids (aa), which are divided into four functional regions: the N‐terminal domain (1‐69aa), the coiled‐coil (CC) domain (70‐94aa), the proline‐rich repeat sequence (P‐X‐X‐P) domain (103‐246aa), and the C‐terminal domain (247‐363aa). We constructed four PSRC1 truncation constructs in addition to the full‐length (FL) construct and overexpressed these constructs in HEK‐293T cells (Figure 9L). Co‐IP analysis revealed that deletion of the P‐X‐X‐P domain (103‐246aa) completely abolished the interaction between PSRC1 and AKT, whereas deletion of other domains had no significant effect on this interaction (Figure 9L). These results indicate that the P‐X‐X‐P domain (103‐246aa) of PSRC1 is essential for its interaction with AKT. Finally, we downloaded the protein structures of PSRC1 and AKT1 from the UniProt database and performed molecular docking simulations using HADDOCK software. The docking results further clarified the specific binding sites between PSRC1 and AKT1, with a binding free energy (ΔG) of −28.4 kcal/mol, indicating a stable interaction (Figure 9M).

In conclusion, our data delineate a precise molecular mechanism: PSRC1 binds to the kinase domain of AKT via its P‐X‐X‐P domain (103‐246aa), thereby competitively inhibiting PTEN‐mediated dephosphorylation of AKT and ultimately activating the AKT/mTOR/HIF‐1α signaling axis to drive Wilms tumor progression.

### 3.7 HIF‐1α Acts as a Transcription Factor to Directly Activate PSRC1 Transcription and Form a Positive Feedback Loop

**FIGURE 10:**
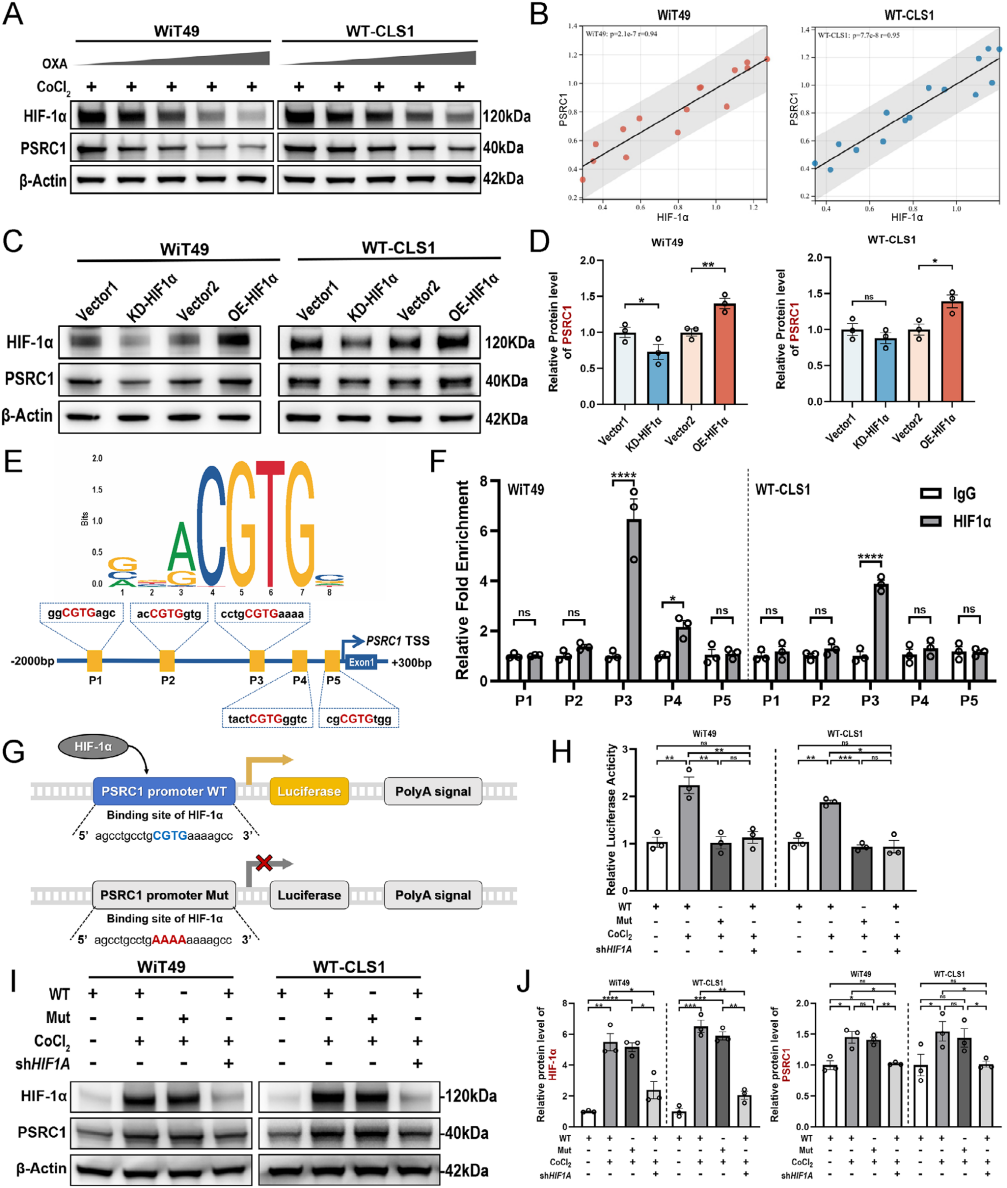
HIF‐1α Binds to the PSRC1 Promoter and Activates Its Transcription. (**A, B)** WB images (A) and Pearson correlation analysis (B) of HIF‐1α and PSRC1 protein levels in WiT49 and WT‐CLS1 cells treated with gradient concentrations of oxamate (0, 2.5, 5, 10, 20 mM). (**C, D)** WB images (C) and relative protein levels of PSRC1(D) in WiT49 and WT‐CLS1 cells after KD/OE‐HIF1α under hypoxic conditions (Vector1 vs. KD‐HIF1α; Vector2 vs. OE‐HIF1α; *n* = 3 per group). (**E)** JASPAR database prediction results showing potential HIF‐1α binding sequences and sites in the PSRC1 promoter region. (**F)** ChIP‐qPCR results pinpointing the specific HIF‐1α binding sites (P3) in the PSRC1 promoter region. (**G)** Schematic of wild‐type (promoter WT) and HRE‐mutated (Mut) PSRC1 promoter luciferase reporter constructs. (**H)** Dual‐luciferase reporter assay under four conditions (*n* = 3). (**I, J)** WB images (I) and relative HIF‐1α and PSRC1 protein levels (J) under the four conditions. Data are presented as mean ± SD. ^*^ *p* < 0.05, ^**^ *p* < 0.01, ^***^ *p* < 0.001, ^****^ *p* < 0.0001.

Finally, we explored the upstream regulatory mechanism by which histone lactylation, particularly H3K18la, activates PSRC1 transcription. In our previous mechanistic studies, we identified HIF‐1α as a key downstream effector of the PSRC1/AKT/mTOR axis. Notably, HIF‐1α is a well‐characterized transcription factor that drives the expression of numerous pro‐tumorigenic genes across multiple cancer types [32]. Moreover, HIF‐1α has been reported to upregulate the expression and activity of glycolysis‐related proteins and is highly expressed in Wilms tumor tissues [33, 34]. Consistently, treatment of Wilms tumor cell lines with gradient concentrations of oxamate resulted in a positively correlated change in the protein expression levels of HIF‐1α and PSRC1 (Figure 10A,B; Figure S6A,B). Based on these observations, we hypothesized that in Wilms tumor, HIF‐1α might function as a transcription factor to bind the PSRC1 promoter region and synergize with H3K18la to enhance PSRC1 transcription.

To verify this hypothesis, we manipulated HIF‐1α expression in Wilms tumor cells under hypoxic conditions. The results showed that HIF‐1α knockdown (KD‐HIF1α) significantly inhibited PSRC1 expression, whereas HIF‐1α overexpression (OE‐HIF1α) enhanced PSRC1 levels (Figure 10C,D; Figure S6C). As a transcription factor, HIF‐1α typically activates target gene transcription by binding to the core hypoxia response element (HRE) sequence 5'‐CGTG‐3' within gene promoters [35]. To identify potential HREs in the PSRC1 promoter, we analyzed the PSRC1 promoter sequence using the JASPAR database, which predicted multiple putative HIF‐1α binding sites (Figure 10E). We then designed five pairs of specific primers targeting these predicted sites and performed ChIP‐qPCR assays, which further pinpointed the exact HIF‐1α binding regions within the PSRC1 promoter (Figure 10F).

To functionally validate the transcriptional activation of PSRC1 by HIF‐1α, we performed a dual‐luciferase reporter assay using wild‐type (promoter WT) and HRE‐mutated (Mut) PSRC1 promoter constructs, with the Mut containing point mutations at the P3 binding site identified by ChIP‐qPCR (Figure 10G). Instead of HIF‐1α overexpression, which may lead to supraphysiological levels and potential non‐specific binding, we chose to induce endogenous HIF‐1α stabilization using the hypoxia mimetic CoCl_2_. This approach better recapitulates the native tumor microenvironment. The experiment included the following four groups: WT promoter + normoxia, WT promoter + hypoxia, Mut promoter + hypoxia, and WT promoter + hypoxia + shHIF1A. The results showed that relative luciferase activity was significantly increased only in the WT+hypoxia group, whereas hypoxia failed to induce luciferase activity in the mutant promoter group, and HIF‐1α knockdown markedly blunted hypoxia‐induced luciferase activity (Figure 10H). In addition, we performed supplementary Western blot analysis using the same grouping conditions (Figure 10I,J). The results confirmed that HIF‐1α protein was significantly induced in the hypoxia‐treated groups, and PSRC1 protein expression showed a consistent increasing trend under hypoxia, further supporting the transcriptional regulation of PSRC1 by HIF‐1α.

**FIGURE 11:**
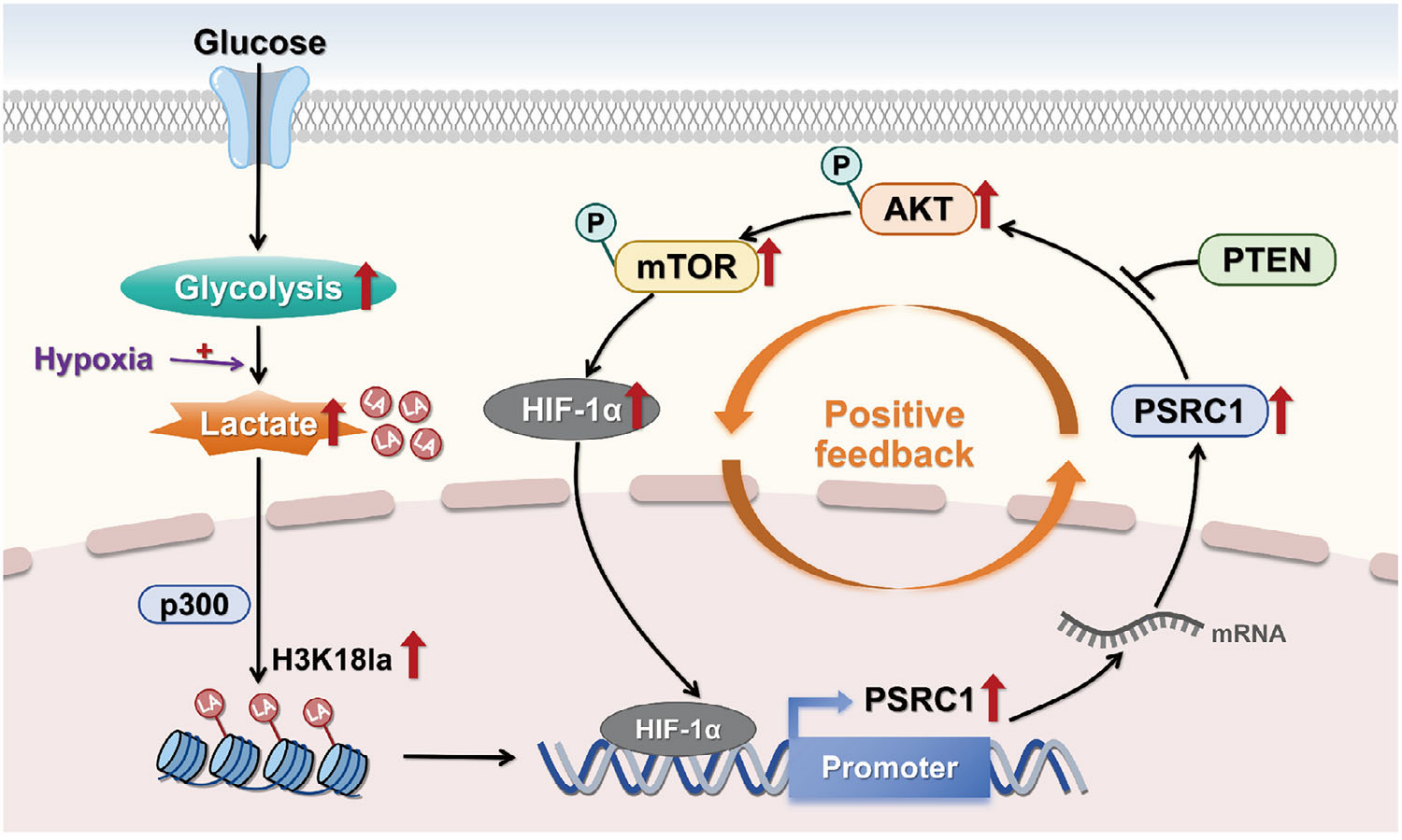
Schematic Diagram of the H3K18la/PSRC1/AKT/mTOR/HIF‐1α/PSRC1 Positive Feedback Loop in Wilms Tumor. Red arrows indicate promotion/activation; T‐bars indicate inhibition.

In summary, our findings reveal a novel positive feedback loop in WT (Figure 11): Histone H3K18 lactylation (H3K18la) promotes PSRC1 transcription, and PSRC1 subsequently activates the AKT/mTOR pathway to enhance HIF‐1α protein stability. In turn, HIF‐1α acts as a transcription factor to directly bind the PSRC1 promoter and further upregulate its expression, which synergizes with H3K18la to accelerate the malignant progression of WT. This newly identified regulatory loop provides promising molecular targets for the diagnosis and targeted therapy of Wilms tumor.

## 4 Discussion

Wilms tumor (WT) is the most common renal malignancy in children. Despite considerable advances in therapeutic strategies, the prognosis of high‐risk and relapsed patients remains dismal, and some survivors are burdened with long‐term treatment‐related sequelae [1, 2, 3]. Over the past six decades, few novel chemotherapeutic agents have been developed for WT. In recent years, tumor molecular profiling has been integrated into the risk stratification system of Children's Oncology Group (COG) trials [36], representing a pivotal step toward precision medicine in WT management. This study bridges metabolic reprogramming, epigenetic modification, and key signaling pathway activation, and for the first time identifies a positive feedback regulatory circuit in WT formed by histone lactylation modification and the PSRC1/AKT/HIF‐1α axis it regulates. These findings provide a novel perspective for understanding the malignant progression of WT and offer potential therapeutic strategies.

Our study revealed prominent metabolic reprogramming in Wilms tumor, characterized by aberrantly activated glycolysis that drives substantial lactate accumulation in the tumor microenvironment. This accumulated lactate serves as a critical substrate for epigenetic modification, leading to a global increase in histone lysine lactylation (Kla) levels, with a particularly significant upregulation of H3K18 lactylation (H3K18la). In vitro cellular functional assays confirmed that modulating histone lactylation levels can alter the proliferative and migratory capacities of WT cells, establishing the tumor‐promoting role of histone lactylation, especially H3K18la. This finding is consistent with previous reports that lactylation facilitates tumor progression in various other cancer types [17] and addresses the research gap regarding histone lactylation in the context of Wilms tumor.

Through the integration of public database mining and experimental validation, we successfully identified PSRC1 as a key downstream effector target of H3K18la. PSRC1 (Proline/Serine‐Rich Coiled‐Coil Protein 1), also known as DDA3, is a crucial mitotic regulator whose normal function is essential for orderly cell cycle progression. Meanwhile, PSRC1 has been identified as an oncogene downregulated by p53 and DNA damage [37], and its oncogenic potential has been verified in multiple tumor types. For example, it is a core risk gene in small cell lung cancer [38], drives tumor growth and metastasis in breast cancer [39], and acts as a hub gene linking glycolysis to immunosuppression in hepatocellular carcinoma [40]. This study further expands its oncogenic profile, demonstrating that PSRC1 is highly expressed in WT, regulated by lactate levels and the writer enzyme p300, and correlates with poor prognosis. In vitro and in vivo functional experiments confirmed its role in promoting tumor growth and migration. Furthermore, PSRC1 overexpression could partially reverse the anti‐tumor effects of glycolysis inhibitors. These results suggest that PSRC1 is not only a prognostic biomarker but also a potential target for overcoming resistance to metabolic intervention.

Mechanistic exploration constitutes the core of this study. First, gene set enrichment analysis (GSEA) revealed that the AKT/mTOR and HIF‐1α signaling pathways were among the most significantly enriched pathways in the PSRC1 high‐expression group, implying that PSRC1 may exert its oncogenic effects by regulating this key signaling axis. Notably, numerous studies have confirmed that the AKT/mTOR signaling pathway drives tumor progression by regulating HIF‐1α protein stability. In pancreatic cancer, ITGA3 activates the FAK/PI3K/AKT/mTOR signaling pathway in an autocrine manner, thereby upregulating the expression of HIF1α, a critical glycolysis regulator [41]. In gastric cancer, the EIF2S2 and KIRREL genes transcriptionally upregulate HIF‐1α by activating the PI3K/AKT/mTOR pathway, driving cancer progression [42, 43]. Consistently, PSRC1 manipulation specifically altered AKT/mTOR phosphorylation and HIF‐1α protein stability, confirming that PSRC1 exerts its oncogenic effect partially via the AKT/mTOR/HIF‐1α axis. HIF‐1α can drive metabolic reprogramming by promoting glycolysis via the Warburg effect, facilitating the growth of various tumor cells [33]. Thus, we have preliminarily delineated a clear downstream signaling axis from PSRC1 to HIF‐1α.

PTEN (Phosphatase and Tensin Homolog) is the canonical negative regulator of AKT, indirectly inactivating AKT by dephosphorylating PIP3 [44]. PTEN functions as a tumor suppressor in various cancer types. In ovarian cancer, PTEN overexpression significantly reduces p‐AKT protein levels and inhibits tumor progression [45]; In pediatric acute lymphoblastic leukemia, PTEN loss leads to dysregulation of the PI3K/AKT pathway, mediating chemotherapy resistance [46]; In drug mechanism studies, statins have been shown to increase PTEN expression through multiple pathways, reducing AKT phosphorylation, thereby promoting apoptosis and inhibiting tumor cell migration and proliferation [47]. Recent studies have revealed that PTEN not only indirectly inhibits AKT through its classical lipid phosphatase activity but also directly binds to AKT as a protein phosphatase, directly suppressing AKT kinase activity and bypassing the classical PTEN‐PIP3‐PDK1‐AKT signaling pathway [29]. Studies have confirmed that AKT interacts with the C2 domain of PTEN via its central kinase domain, and PTEN can target AKT to suppress tumorigenesis through its protein phosphatase activity [29].

In this study, through mass spectrometry (MS), exogenous and endogenous co‐immunoprecipitation (Co‐IP), and molecular docking analysis, we confirmed a direct protein‐protein interaction between PSRC1 and AKT. Co‐IP and truncation experiments demonstrated that the 103–246aa domain of PSRC1 directly binds to the central kinase domain of AKT. Upregulating or downregulating PSRC1 expression decreased or increased the binding between PTEN and AKT, respectively. Therefore, we conclude that PSRC1 increases AKT phosphorylation levels by competitively binding to the central kinase domain of AKT with PTEN, thereby promoting the sustained activation of downstream pathways. A similar “competitive binding” regulatory model has been reported in other tumors. For example, in bladder cancer, CDCA8 was shown to maintain HIF‐1α protein stability by competitively binding to AKT against PTEN [30]. In pancreatic cancer, DUSP2 competes with AKT1 to bind the casein kinase 2 alpha subunit (CSNK2A1), inhibiting AKT1 phosphorylation and thus suppressing tumor progression [31]. Additionally, Capivasertib, an FDA‐approved novel drug, inhibits AKT phosphorylation by competing with ATP for binding to AKT and is currently used in breast cancer treatment [48]. Interestingly, the recurrent employment of this “competitive binding” strategy by mitosis‐related proteins (such as CDCA8, CSNK2A1, and PSRC1) across different tumor types suggests that certain mitotic proteins may act as critical molecular bridges, functionally coupling the high‐speed cell division program with AKT‐driven pro‐survival signaling pathways to synergistically promote unlimited tumor proliferation.

Activation of the AKT/mTOR pathway further stabilizes and increases the protein levels of its key downstream effector, HIF‐1α. As a core transcription factor for cellular adaptation to hypoxia and metabolic remodeling, the accumulation of HIF‐1α feedback enhances glycolysis and lactate production by upregulating genes such as GLUT1 and LDHA [33, 49]. Another innovative finding of this study is that through bioinformatic prediction and ChIP‐qPCR experiments, we discovered that HIF‐1α can directly bind to the promoter region of the PSRC1 gene to activate its transcription, thereby forming a positive feedback loop. Numerous studies have confirmed that HIF‐1α can form feedback loops with the AKT/mTOR signaling pathway through its downstream target genes, maintaining high HIF‐1α expression and promoting malignant tumor progression. For example, the HIF‐1α/HIF‐2α‐EGF/EGFR‐PI3K/AKT‐mTOR‐HIF1α signaling axis promotes glioblastoma growth through a positive feedback mechanism [50]; in esophageal squamous cell carcinoma, Claudin‐1/4, as direct targets of HIF‐1α, feedback‐regulate HIF‐1α expression via the PI3K‐AKT‐mTOR pathway [51]. A study on anaplastic Wilms tumor found that IGF1 upregulates HIF‐1α and its downstream target IGFBP2 via the IGF1R‐AKT pathway, while HIF‐1α and IGFBP2 further positively enhance IGF1‐AKT signaling, forming a positive feedback vicious cycle that drives tumor growth and metastasis [52]. Our findings further refine the network of feedback mechanisms in Wilms tumor.

Currently, there are no approved drugs specifically targeting histone lactylation, with most therapeutic strategies remaining in preclinical stages [53]. Here, we focus on three promising approaches relevant to our findings. First, inhibiting lactate production. Oxamate, a classic LDHA inhibitor, has shown antitumor activity and low toxicity across multiple preclinical cancer models [54]; although its clinical translation is limited by high polarity and poor membrane permeability, its hydrophilic nature facilitates loading into lipid‐based nanoparticles and the design of nanocomplexes that can carry additional antineoplastic drugs for release within the tumor microenvironment [54]. Second, targeting the “writer” p300: p300/CBP inhibitors are classified into HAT inhibitors and bromodomain inhibitors, with CCS1477 (inobrodib) having advanced to phase I/II clinical trials for multiple myeloma and prostate cancer [55]. Third, disrupting the PSRC1/AKT/mTOR/HIF‐1α positive feedback loop, which can be achieved through AKT/mTOR inhibitors (e.g., everolimus), tumor‐specific peptide inhibitors targeting the PSRC1‐AKT protein interaction interface, or HIF‐1α inhibitors, particularly effective against large solid tumors with central hypoxic regions. Nevertheless, these strategies still face critical clinical limitations, including the systemic toxicity of certain inhibitors, stricter safety requirements for pediatric patients, and the risk of drug resistance from single‐target inhibition due to the positive feedback loop. These challenges underscore the need for further therapeutic optimization and combination regimen development.

This study also has several limitations. First, the clinical sample size is relatively small, and the value of PSRC1 as an independent prognostic marker requires validation in larger‐scale, prospective cohorts. Second, although we verified the functionality of this axis in cell lines and BALB/c nude mouse xenograft models, intervention studies in more clinically relevant models, such as genetically engineered animal models, have not yet been conducted. Third, our study establishes p300 as the critical ‘writer’ catalyzing H3K18la deposition at the PSRC1 promoter, while the identity and role of the corresponding ‘erasers’ in Wilms tumor remain open questions. Recent studies have identified class I histone deacetylases (HDAC1‐3) and sirtuins (SIRT1‐3) as potential lysine delactylases [18, 19, 20]. It is possible that dysregulation of these eraser enzymes contributes to the aberrant accumulation of H3K18la observed in WT. Given that histone lactylation and acetylation share enzymatic machineries, their potential mechanistic crosstalk remains to be elucidated. A comprehensive investigation of the complete histone lactylation regulatory network in Wilms tumor, including writers, erasers, and readers, as well as its interplay with histone acetylation, will be the focus of our future independent work.

In summary, this study systematically reveals a novel mechanism: ′Histone H3K18 lactylation transcriptionally activates PSRC1, which then competitively binds to AKT against PTEN, activating the AKT/mTOR/HIF‐1α signaling axis. Meanwhile, HIF‐1α feedback enhances PSRC1 transcription, thereby forming a positive feedback loop that drives the malignant progression of Wilms tumor (Figure 11). This discovery not only deepens our understanding of Wilms tumor pathogenesis but, more importantly, identifies multiple nodes within this loop as potential therapeutic targets. Our research provides an important theoretical basis for the precise stratification and individualized treatment of Wilms tumor.

## Author Contributions

**Yanping Wang**: data curation, formal analysis, investigation, methodology, software, validation, visualization, writing – original draft. **Hongjie Gao**: Conceptualization, funding acquisition, Investigation, methodology, resources. **Bifei Zhang**: investigation, validation, writing – review & editing. **Xuetian Li**, **Ding Li,** and **Aihua Cao**: Investigation, validation. **Fengyin Sun**: conceptualization, funding acquisition, methodology, project administration, resources, supervision.

## Conflicts of Interest

The authors declare no conflicts of interest.

## Supporting information




**Supporting File 1**: advs76579‐sup‐0001‐SuppMat.pdf.

**Supporting File 2**: advs76579‐sup‐0002‐Tables.docx.

## Data Availability

The data that support the findings of this study are openly available in GEO (Gene Expression Omnibus) at https://www.ncbi.nlm.nih.gov/geo/, reference number GSE197047/c/156675/295142/245990/304259.
