# Supplementary material for: Histone H3K18 Lactylation Promotes the Malignant Progression of Wilms Tumor via a PSRC1/AKT/HIF‐1α Positive Feedback Loop
Source: Adv Sci (Weinh). 2026 Jul 13;13(56):e76579. doi: 10.1002/advs.76579 (PMC13360103; doi:10.1002/advs.76579)
Supplement: Supplementary file 1 [file ADVS-13-e76579-s001.pdf]

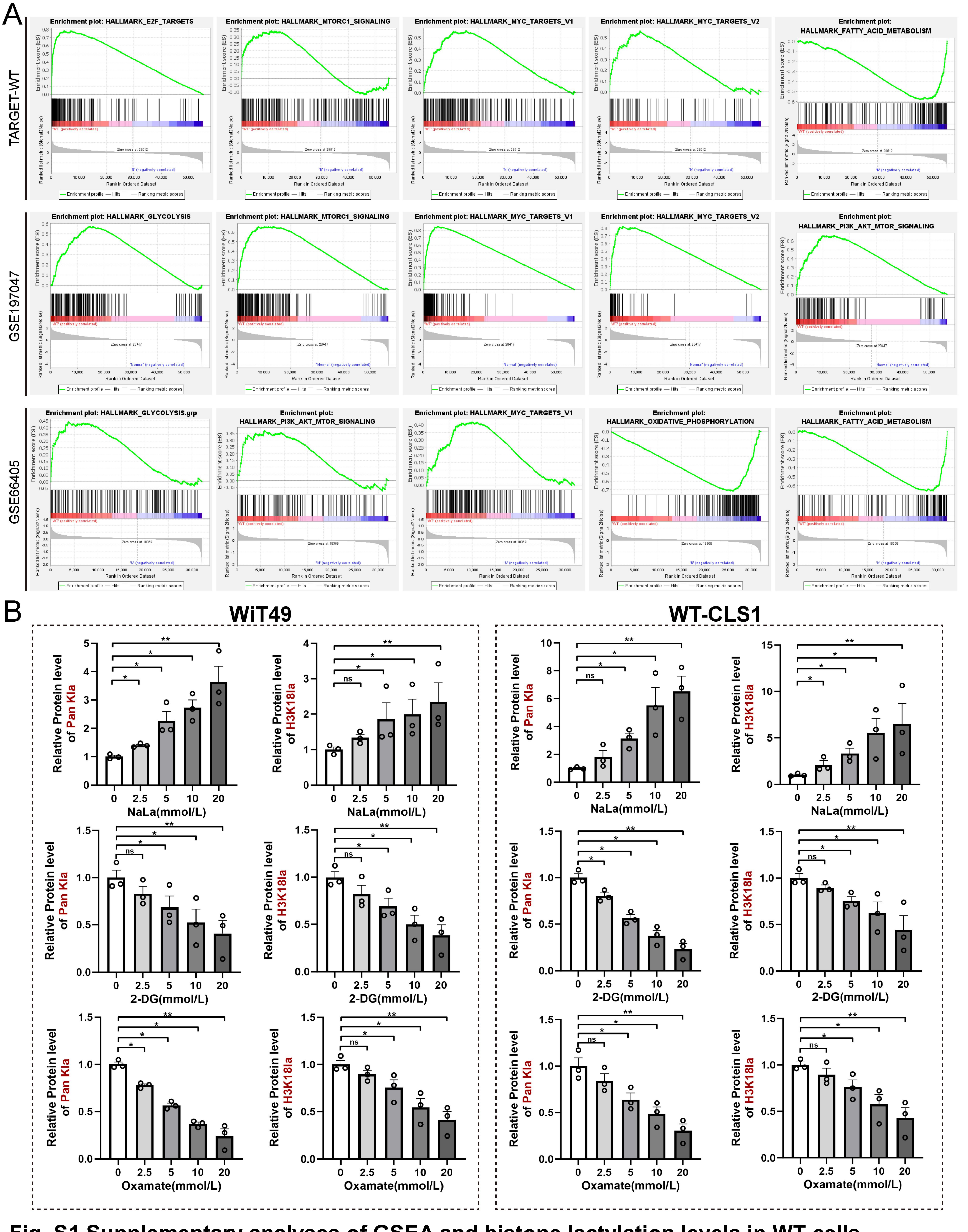

Fig. S1 Supplementary analyses of GSEA and histone lactylation levels in WT cells

A. Supplementary GSEA results of glycolysis-related pathways between WT and normal (N) groups using TARGET database and GEO database (p<0.05). **B.** Quantitative analysis of WB data showing relative protein levels of Pan Kla and H3K18la in WiT49 and WT-CLS1 cells treated with gradient doses of sodium lactate (NaLa), 2-deoxy-D-glucose (2-DG) or oxamate (0, 2.5, 5, 10, 20 mM). Data are presented as mean ± SD. *p<0.05, **p<0.01, ***p<0.001.

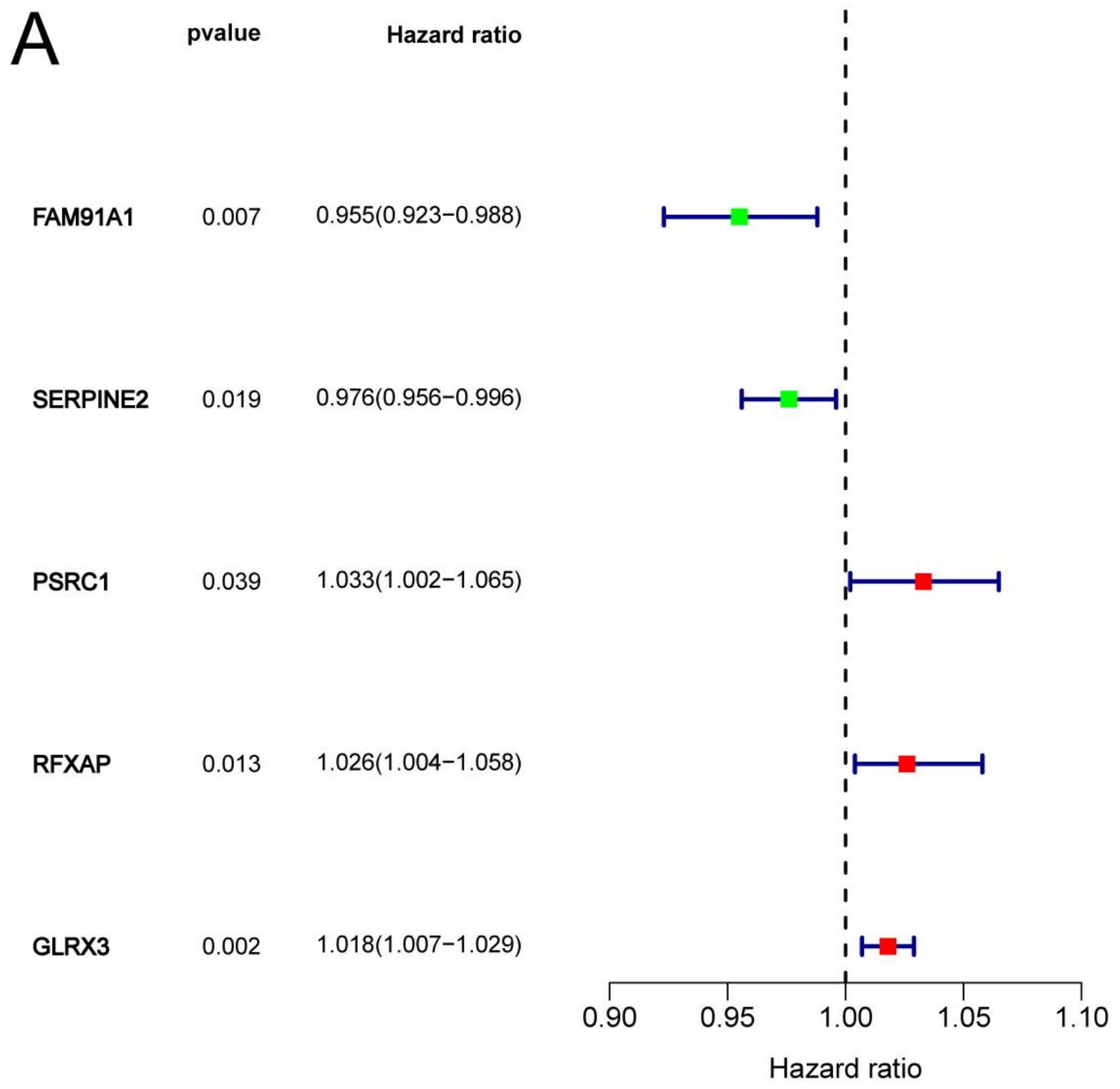

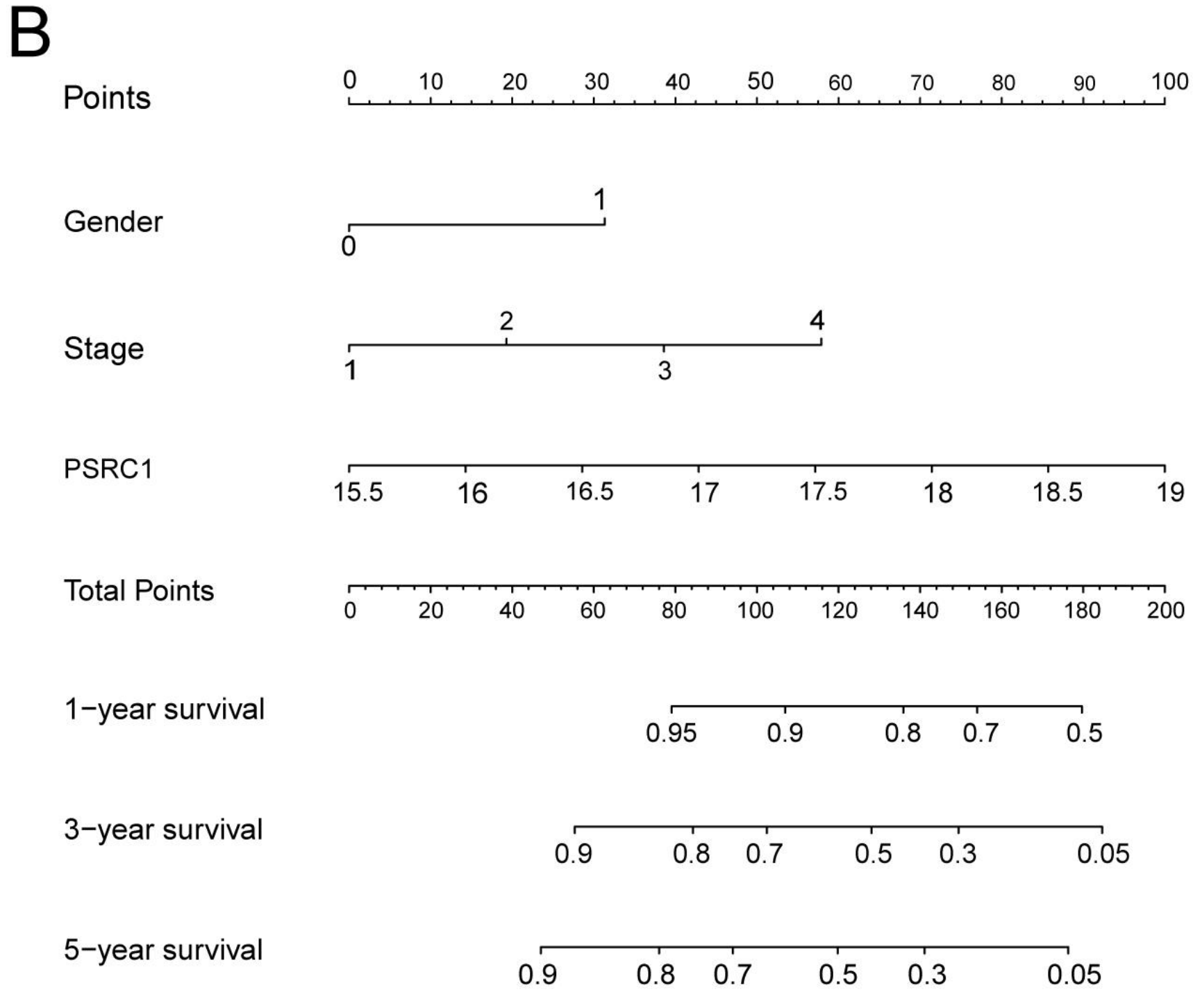

Fig. S2 Prognostic factors and nomogram for overall survival in Wilms tumor

A. Forest plot of multivariate Cox regression analysis (including 10 genes identified by univariate Cox regression), demonstrating that high expression of PSRC1, RFXAP, and GLRX3 serves as independent prognostic factors. **B.** Nomogram depicting the association between gender, clinical stage, PSRC1 expression, and the probability of 1-year, 3-year, and 5-year overall survival (OS) in WT patients.

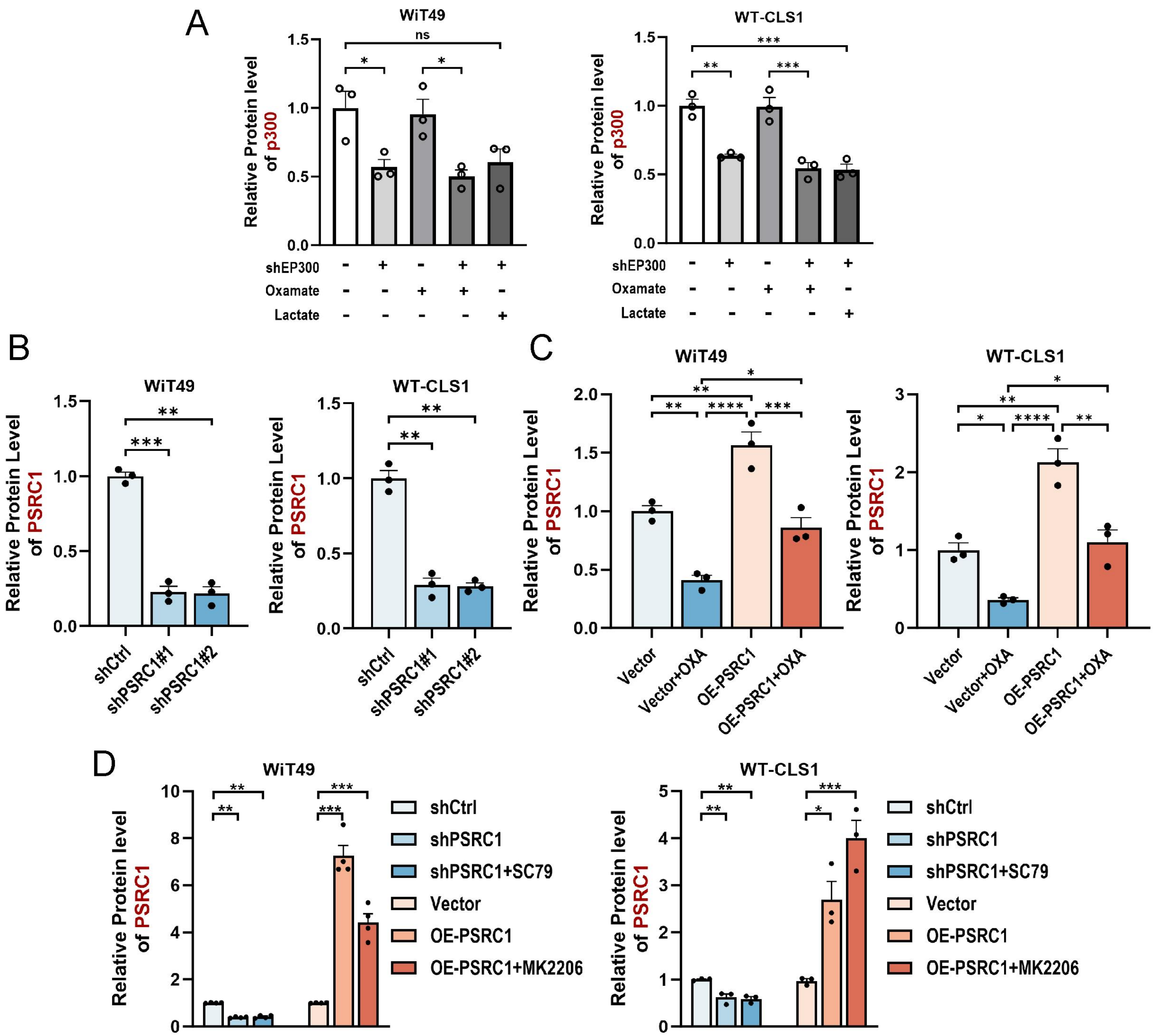

Fig. S3 WB quantitative analysis to evaluate the knockdown / overexpression efficiency

A. WB quantitative analysis of relative p300 protein levels in WiT49 and WT-CLS1 cells to evaluate the knockdown efficiency of shEP300 groups (n=3 per group). **B-D.** WB quantitative analysis of relative PSRC1 protein levels in WiT49 and WT-CLS1 cells to evaluate the knockdown / overexpression efficiency of shPSRC1 / OE-PSRC1 groups (n=3 per group). Data are presented as mean ± SD. *p<0.05, **p<0.01, ***p<0.001, ****p<0.001.

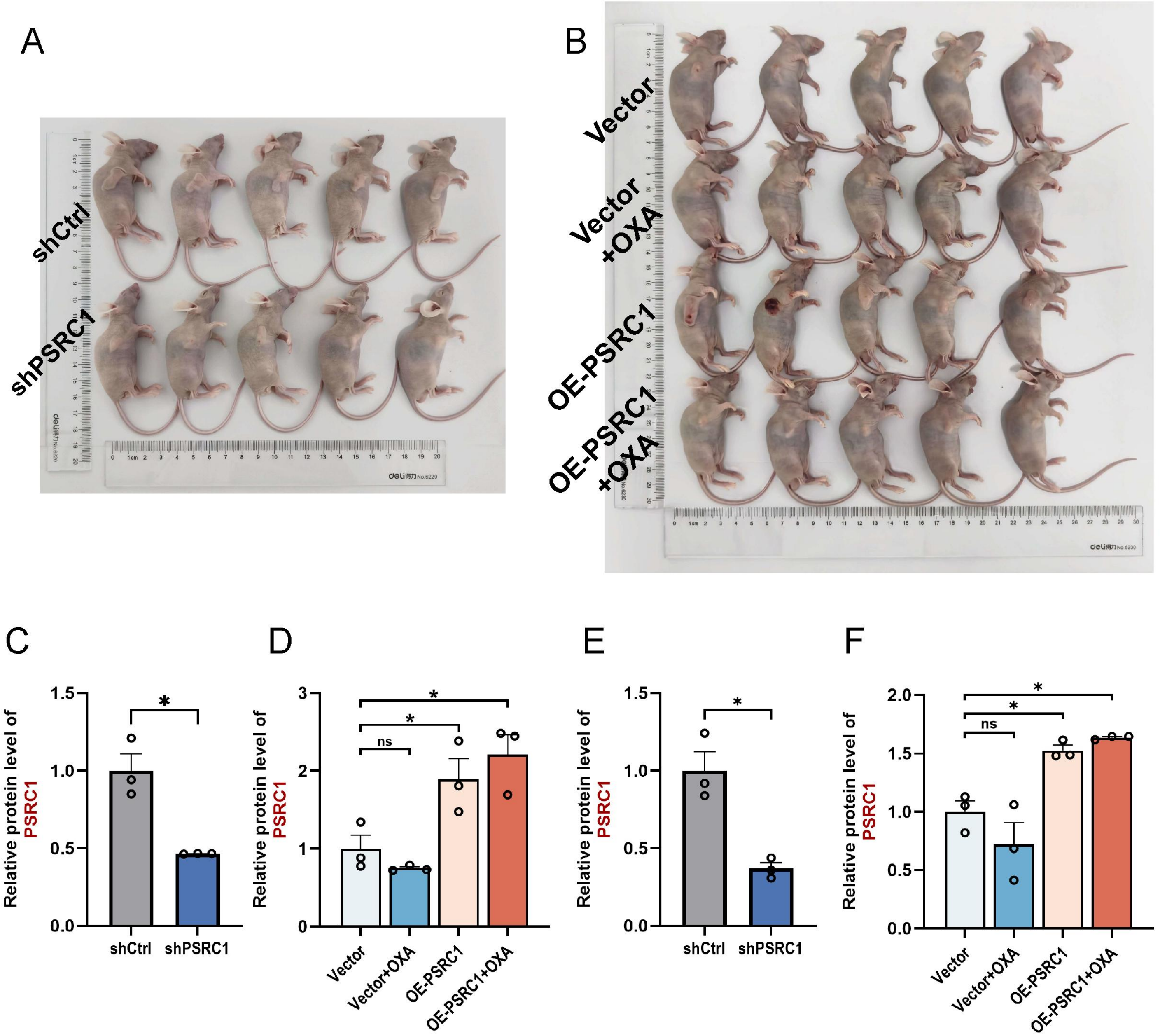

Fig. S4 Gross images of subcutaneous xenograft-bearing mice and Western blot validation of PSRC1 expression in xenograft tumor tissues

A. Images of BALB/c nude mice xenograft models prior to dissection in shCtrl and shPSRC1 groups (n=5 per group). **B.** Images of BALB/c nude mice xenograft models prior to dissection in Vector, Vector+Oxamate (OXA), OE-PSRC1 and OE-PSRC1+OXA groups (n=5 per group). **C.** WB validation of PSRC1 knockdown in xenograft tumor tissues (supplementary to Fig. 8E). **D.** WB validation of PSRC1 overexpression in xenograft tumor tissues (supplementary to Fig. 8F). **E.** WB validation of PSRC1 knockdown in xenograft tumor tissues (supplementary to Fig. 8G). **F.** WB validation of PSRC1 overexpression in xenograft tumor tissues (supplementary to Fig. 8H). Data are presented as mean ± SD. *p<0.05, **p<0.01, ***p<0.001.

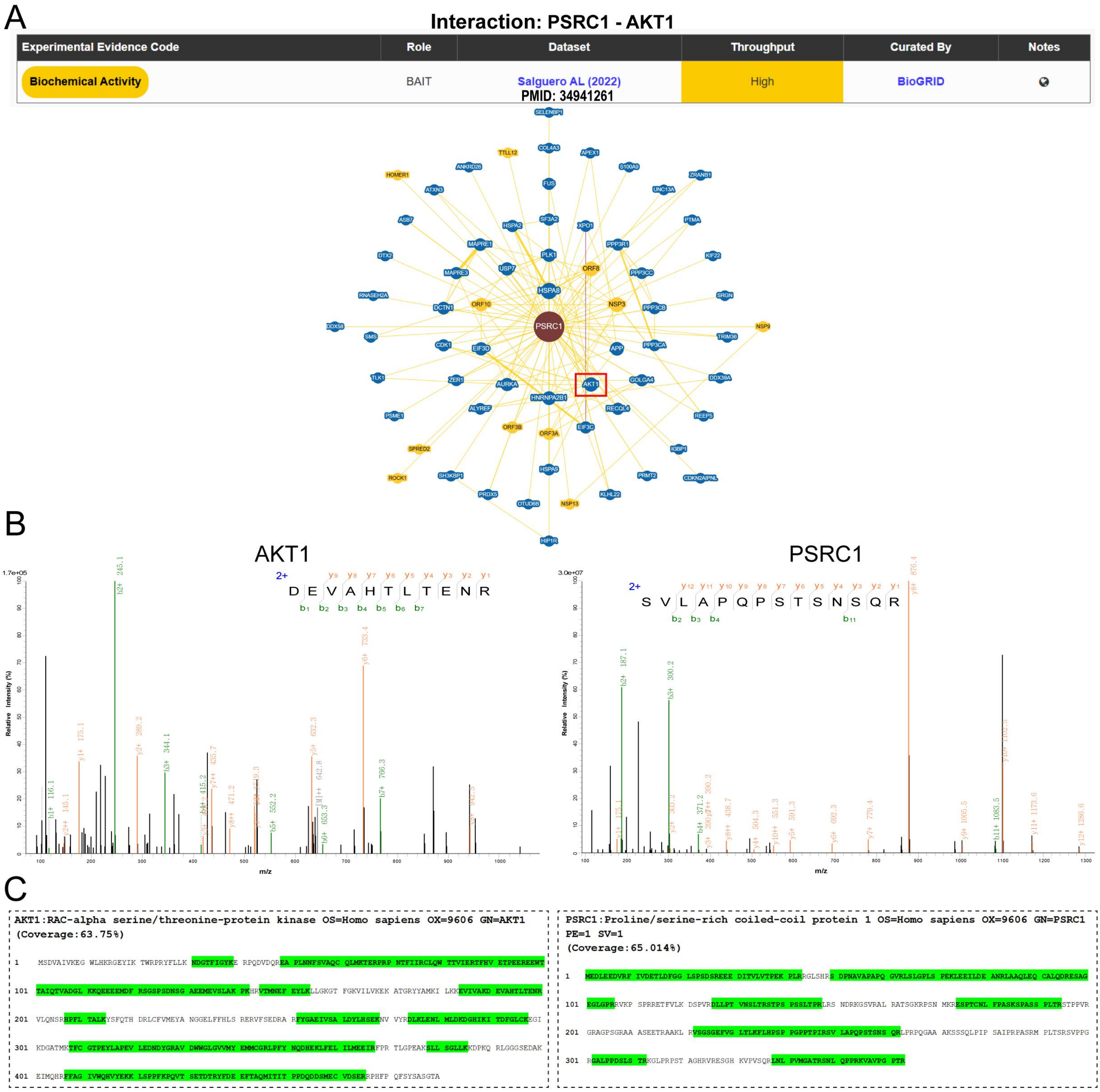

Fig. S5 Prediction and Mass Spectrometry (MS) validation of PSRC1-interacting proteins

A. BioGRID database prediction results of potential PSRC1-interacting proteins. **B**. MS/MS spectra of AKT1 and PSRC1 proteins identified from the PSRC1-IP protein complex. **C.**Protein sequence coverages of AKT1 and PSRC1 identified by MS analysis of the PSRC1-IP protein complex.

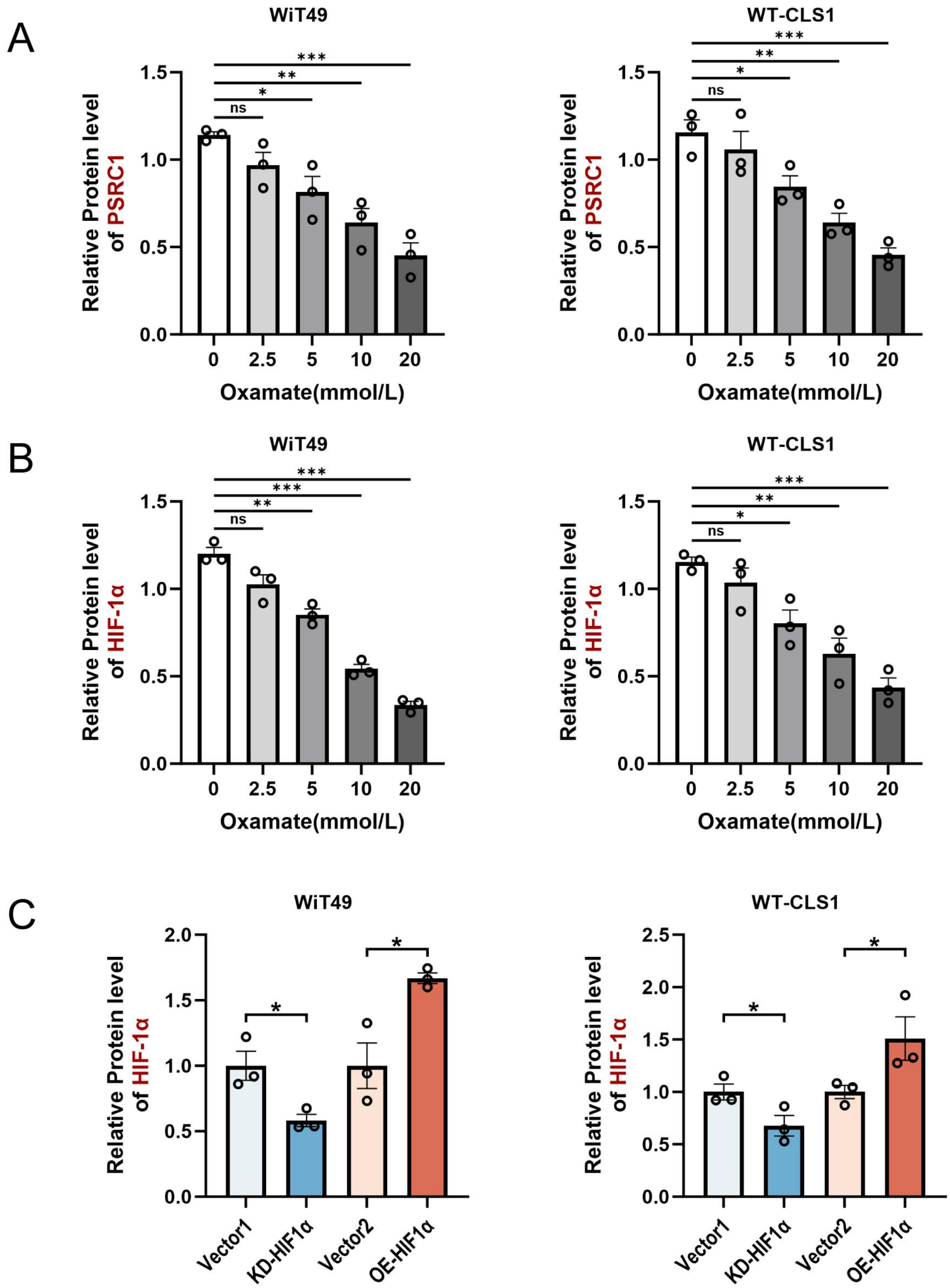

Fig. S6 Validation of HIF-1α regulation of PSRC1 expression

A-B. Supplementary WB quantitative results of relative protein levels of PSRC1 (A) and HIF-1α (B) in WiT49 and WT-CLS1 cells treated with gradient concentrations of oxamate (0, 2.5, 5, 10, 20 mM). **C.** WB quantitative analysis of relative HIF-1α protein levels in WiT49 and WT-CLS1 cells to evaluate the knockdown/overexpression efficiency of KD/OE-HIF1α groups under hypoxic conditions (n=3 per group). Data are presented as mean ± SD. *p<0.05, **p<0.01, ***p<0.001.
