# Supplementary material for: Histone H3K18 Lactylation Promotes the Malignant Progression of Wilms Tumor via a PSRC1/AKT/HIF‐1α Positive Feedback Loop
Source: Adv Sci (Weinh). 2026 Jul 13;13(56):e76579. doi: 10.1002/advs.76579 (PMC13360103; doi:10.1002/advs.76579)
Supplement: Supplementary file 2 [file ADVS-13-e76579-s002.docx]

**Supplementary Tables**

**Table S1. The sequences of shRNA used in this study**

| **shRNA** | **Sequence (5'-3')** |
| --- | --- |
| shPSRC1#1 | GCATTGGTTCACTGGACATTT |
| shPSRC1#2 | TCCCAGGACCTACCAGGTAAA |
| shPTEN | CTAGAACTTATCAAACCCTTT |
| shEP300#1 | CAATTCCGAGACATCTTGAGA |
| shEP300#2 | CCCGGTGAACTCTCCTATAAT |
| shHIF1A | CCGCTGGAGACACAATCATAT |

**Table S2. Primers for RT-qPCR used in this study**

| **Gene Symbol** | **Coding Protein** | **Primer Sequences（5'-3'）** |
| --- | --- | --- |
| ***PSRC1*** | Proline and serine rich coiled-coil 1 | **F:** ACTAGGTTGCGGGAGAAAGC  **R:** TTAGATCTCCAGCCCACCCT |
| ***RFXAP*** | Regulatory factor X associated protein | **F:** ACGTCAAACTCGAGGAAAGTG  **R:** TGCAGGACGATCCCCAAAAG |
| ***GLRX3*** | Glutaredoxin 3 | **F:** TGGATTGCTTCTGTCTGGCG  **R:** GACCACAAGGAGGGACTTGG |
| ***HIF1A*** | hypoxia inducible factor 1 subunit alpha (HIF-1α) | **F:** AGAGGTTGAGGGACGGAGAT  **R:** GACGTTCAGAACTTATCCTACCAT |
| ***ACTB*** | Actin beta (β-Actin) | **F:** TGCGTGACATTAAGGAGAAG  **R:** CTGCATCCTGTCGGCAATG |

‘F’ represents ‘forward primers’, and ‘R’ represents ‘reverse primers’.

**Table S3. Primers for ChIP-qPCR used in this study**

| **ChIP Target**  **(Antibody)** | **Coding Protein** | **Primer Sequences（5'-3'）** |
| --- | --- | --- |
| H3K18la-IP | ***a*** | **F:** AAGCAGTGAGGATGACCAGC  **R:** GAACTTGGGAGGCAGAGCTT |
| H3K18la-IP | ***b*** | **F:** AGTACAGACGGGGTTTCACC  **R:** GCAGTAAAGAAGCCGGCCAA |
| H3K18la-IP | ***c*** | **F:** CCTACTCCAGAGGCAGAGCA  **R:** GTTCCAGGGAGTTGCCTACC |
| H3K18la-IP | **d** | **F:** AAACCGTGGTGATGCTGTCT  **R:** ACCGAAACCAAGATGGCGAT |
| H3K18la-IP | **e** | **F:** ATACAGCCCAGGTCTCAACC  **R:** GGCCACCAGTGAACTTGTCA |
| H3K18la-IP | **f** | **F:** AACTGCGAGGACCAACATCC  **R:** CAAGTTTCGGTTTAGAGGCGG |
| H3K18la-IP | **g** | **F:** GTGCGCCCAAGAAAACGAAT  **R:** GAGCACCCAGTCTCCAGAAC |
| H3K18la-IP | **h** | **F:** CTCTGCTCCCATCTGCCTAC  **R:** TTCCCAGAAGACTGCATCCC |
| H3K18la-IP | **i** | **F:** GGGACTAGGTTGCGGGAGAA  **R:** CCACTATCCTCAGGGTTCTGC |
| H3K18la-IP | **j** | **F:** CGGAGGTAGCAGGAGTAGCT  **R:** AGGCTCGCAGCTAGATTCAG |
| p300-IP | **a** | **F:** CGGGAAAGGGGTAGGCAAC  **R:** CACTCCAGACAGCATCACCA |
| p300-IP | **b** | **F:** ACACCCCCTCTAAATCCCCT  **R:** TGTTGGTCCTCGCAGTTACG |
| p300-IP | **c** | **F:** GCATTTGAACCTAAGCCTGCC  **R:** CATCCTCCATCGCCTCTTCA |
| p300-IP | **d** | **F:** GTTCTGGAGACTGGGTGCTC  **R:** CAGGAGGGAAGAAACGGACC |
| p300-IP | **e** | **F:** GGGATGCAGTCTTCTGGGAA  **R:** CCCACCTACTCAAGCCACAC |
| p300-IP | **f** | **F:** GAGTGCAGAACCCTGAGGAT  **R:** GAACGATACGGAGGGCTCC |
| HIF1α-IP | **P1** | **F:** TGGATCTCCTGACCTCGTGA  **R:** GCCAAATCTGCCAGAAACCA |
| HIF1α-IP | **P2** | **F:** TGTAAACCGTGGTGATGCTG  **R:** TGGCGATGAAACTGACCTCT |
| HIF1α-IP | **P3** | **F:** CCGCCTCTAAACCGAAACTTG  **R:** GGTGCAAAGAAAGCAACGGG |
| HIF1α-IP | **P4** | **F:** GTTCTGGAGACTGGGTGCTC  **R:** GTAGGCAGATGGGAGCAGAG |
| HIF1α-IP | **P5** | **F:** CCCCACCGGCTGACTTCC  **R:** CGGGGCTCCTTTCGAATCTC |

**Notes:** All ChIP-qPCR target regions are the promoter region of *PSRC1* (-2000bp ~ +300bp); ‘F’ represents ‘forward primers’, and ‘R’ represents ‘reverse primers’.

**Table S4. Antibodies used in this study**

| **Antibody** | **Source** | **Identifier** | **Dilution (Application)** |
| --- | --- | --- | --- |
| Anti-L-Lactyl Lysine Rabbit mAb | PTM Bio | Cat# PTM-1401RM  RRID: AB_2942013 | 1:1000 (WB)  1:200 (IHC) |
| Anti-Lactyl-Histone H3 (Lys18) Rabbit mAb | PTM Bio | Cat# PTM-1406RM  RRID: AB_2909438 | 1:1000 (WB)  1:200 (IHC) |
| Anti-Lactyl-Histone H3 (Lys18) Rabbit mAb | PTM Bio | Cat# PTM-1427RM  RRID: AB_3076698 | 6 μg/5x10^6^ cells (ChIP) |
| Anti-L-Lactyl-Histone H3 (Lys9) Rabbit mAb | PTM Bio | Cat# PTM-1419RM  RRID: AB_3076695 | 1:200 (IHC) |
| Anti-L-Lactyl-Histone H4 (Lys12) Rabbit mAb | PTM Bio | Cat# PTM-1411RM  RRID: AB_2941896 | 1:200 (IHC) |
| Anti-Acetyl-Histone H3 (Lys18) Rabbit mAb | PTM Bio | Cat# PTM-114RM  RRID: AB_3714608 | 1:2000 (WB) |
| Anti-Histone H3 Rabbit mAb | PTM Bio | Cat# PTM-1001RM  RRID: AB_3676032 | 1:5000 (WB) |
| Anti-PSRC1 Rabbit pAb | ThermoFisher | Cat# PA5-78606  RRID: AB_2736472 | 1:2000 (WB)  1:200 (IHC)  1:200 (IF)  1:50 (IP) |
| Anti-KAT3B/p300  Rabbit mAb | Abcam | Cat# ab275378  RRID: AB_2935873 | 5 μg/5x10^6^ cells (ChIP)  1:1000 (WB) |
| Anti-DDDDK Tag (Binds to FLAG tag sequence) Rabbit mAb | Abcam | Cat# ab205606  RRID: AB_2916341 | 1:5000 (WB) |
| Anti-Myc Tag Mouse mAb | Abcam | Cat# ab32  RRID: AB_303599 | 1:1000 (WB) |
| Anti-pan-AKT Rabbit pAb | Abcam | Cat# ab8805  RRID: AB_306791 | 1:2000 (WB) |
| Anti-AKT1(pS473)+AKT2(pS474)+AKT3 (pS472) Rabbit mAb | Abcam | Cat# ab192623  RRID: AB_2934087 | 1:1000 (WB) |
| Anti-mTOR Rabbit mAb | Abcam | Cat# ab32028  RRID: AB_881283 | 1:1000 (WB) |
| Anti-mTOR (phosphoS2448) Rabbit mAb | Abcam | Cat# ab109268  RRID: AB_10888105 | 1:1000 (WB) |
| Anti-PTEN (D4.3) Rabbit mAb | Cell Signaling Technology | Cat# 9188  RRID: AB_390810 | 1:1000 (WB)  1:200 (IF)  1:100 (IP) |
| Anti-HA tag Rabbit pAb | Proteintech | Cat# ab9110  RRID: AB_11042321 | 1:5000 (WB) |
| Anti-AKT Mouse mAb | Proteintech | Cat# 60203-2-Ig  RRID: AB_10912803 | 1:200 (IF)  1:100 (IP) |
| Anti-HIF-1 alpha Rabbit pAb | Proteintech | Cat# 20960-1-AP  RRID: AB_10732601 | 1:2000 (WB)  6 μg/5x10^6^ cells (ChIP) |
| Anti-IgG Rabbit pAb | Proteintech | Cat# 30000-0-AP  RRID: AB_2819035 | 5 μg/5x10^6^ cells (ChIP) |
| Anti-IgG Mouse pAb | Proteintech | Cat# B900620  RRID: AB_2883054 | 5 μg/5x10^6^ cells (ChIP) |
| Anti-Beta Actin Mouse mAb | Proteintech | Cat# 66009-1-Ig  RRID: AB_2687938 | 1:20000 (WB) |
| Anti-GAPDH Mouse mAb | Proteintech | Cat# 60004-1-Ig  RRID: AB_2107436 | 1:50000 (WB) |

**Notes: CUT&Tag**, Cleavage Under Targets and Tagmentation; **IHC**, immunohistochemistry; **IP,** immunoprecipitation; **mAb**, monoclonal antibody; **pAb**, polyclonal antibody; **WB**, western blot

**Table S5. Kits and reagents used in this study**

| **Kit/Reagent** | **Source** | **Identifier (Cat#)** |
| --- | --- | --- |
| Glucose Assay Kit | Nanjing Jiancheng Bioengineeering Institute | A154-1-1 |
| Lactic Acid Assay Kit | Nanjing Jiancheng Bioengineeering Institute | A019-2-1 |
| NaLa (Lactic acid sodium) | Sigma | HY-B2227B |
| Oxamate (Sodium Oxamate) | MedChemExpress | HY-W013032A |
| 2-DG (2-Deoxy-D-glucose) | MedChemExpress | HY-13966 |
| CoCl_2_ (Cobalt(II) chloride) | Macklin | C805641 |
| SC79 (AKT activator) | Beyotime | SF2730 |
| MK2206 (AKT inhibitor) | Beyotime | SF2712 |
| CHX (Cycloheximide) | MedChemExpress | HY-12320 |
| Lipo8000 | Beyotime | C0533 |
| 4% PFA (paraformaldehyde) | Beyotime | P0099 |
| Sodium Citrate Antigen Retrieval Solution | Solarbio | [C1032](https://www.solarbio.com/goodsInfo?id=1320) |
| Tris-EDTA Antigen Retrieval Solution | Solarbio | C1038 |
| Endogenous peroxidase blocking solution | ZSGB-BIO | PV-9000 |
| DAB Substrate Kit | ZSGB-BIO | ZLI-9018 |
| Mayer’s Hematoxylin Stain Solution, for IHC | Solarbio | [G1080](https://www.solarbio.com/goodsInfo?id=1219) |
| Neutral Balsam | Solarbio | G8590 |
| Cell Lysis Buffer for Western and IP | Beyotime | P0013J |
| RIPA Lysis Buffer | Beyotime | P0013B |
| PMSF Solution (100mM) | Beyotime | ST507 |
| Phosphorylation-specific protease inhibitor | Servicebio | G2007 |
| 5× SDS-PAGE Loading Buffer | Servicebio | G2075 |
| FuturePAGE™ 4-12% 15 Wells | ACE Biotechnology | ET15412Gel |
| Durapore Membrane, PVDF, 0.45 µm | Millipore | HVLP2932A |
| Durapore Membrane, PVDF, 0.22 µm | Millipore | GVHP29325 |
| TBST Buffer | Servicebio | G0004 |
| Skimmed Milk Powder (Blotting Grade) | Beyotime | P0216 |
| Super Sensitive ECL Luminescence Reagent | MeilunBio | MA0186-1 |
| SteadyPure Quick RNA Extraction Kit | Accurate Biotechnology | AG21023 |
| ABScript III RT Master Mix for qPCR with gDNA remover | ABclonal | RK20429 |
| 2× Universal SYBR Green Fast qPCR Mix | ABclonal | RK21203 |
| BeyoChIP™ Enzymatic ChIP Assay Kit | Beyotime | P2083S |
| BeyoMag™ Magnetic Bead-based PCR/DNA Purification Kit | Beyotime | D0041S |
| Cell Counting Kit-8 (CCK-8) | GLPBIO | GK10001 |
| Corning® Transwell® 24 well plates | Millipore | CLS3422 |
| Anti-Flag Magnetic Beads | MedChemExpress | HY-K0207 |
| Anti-c-Myc Magnetic Beads | MedChemExpress | HY-K0206 |
| Anti-HA Magnetic Beads | MedChemExpress | HY-K0201 |
| Protein A/G Magnetic Beads | MedChemExpress | HY-K0202 |
| BeyoBlue™ Plus Coomassie Brilliant Blue Ultra-Fast Staining Solution | Beyotime | P0003 |
| Glass bottom cell culture dish | NEST | 801001 |
| Immunostaining Fixative | Beyotime | P0098 |
| Anti-Fade Mounting Medium with DAPI | Beyotime | P0131 |
| Matrix-Gel™ Basement Membrane Matrix | Beyotime | C0372 |
| DMEM(1×), Dulbecco’s Modified Eagle Medium | MeilunBio | MA0212 |
| Penicillin/Streptomycin, sterile | MeilunBio | PWL062 |
| Fetal Bovine Serum | Procell system | 164210 |
| Dual Luciferase Reporter Gene Assay Kit | Yeasen Biotechnology | 11402ES60 |
